# Creation of Environmentally Friendly Super “Dinitrotoluene Scavenger” Plants

**DOI:** 10.1002/advs.202303785

**Published:** 2023-09-15

**Authors:** Jian‐Jie Gao, Zhen‐Jun Li, Bo Zhu, Li‐Juan Wang, Jing Xu, Bo Wang, Xiao‐Yan Fu, Hong‐Juan Han, Wen‐Hui Zhang, Yong‐Dong Deng, Yu Wang, Zhi‐Hao Zuo, Ri‐He Peng, Yong‐Sheng Tian, Quan‐Hong Yao

**Affiliations:** ^1^ Biotechnology Research Institute Shanghai Academy of Agricultural Sciences Shanghai 201106 China; ^2^ Shanghai Key Laboratory of Agricultural Genetics and Breeding Shanghai Academy of Agricultural Sciences Shanghai 201106 China; ^3^ Key Laboratory for Safety Assessment (Environment) of Agricultural Genetically Modified Organisms Ministry of Agriculture and Rural Affairs Shanghai 201106 China; ^4^ Anhui Province Key Laboratory for the Conservation of Biological Resources College of Life Sciences Anhui Normal University Wuhu 241000 China

**Keywords:** 2,4‐dinitrotoluene (2,4‐DNT), carbon neutrality, complete degradation, rapid phytoremediation, sustainable development goals

## Abstract

Pervasive environmental contamination due to the uncontrolled dispersal of 2,4‐dinitrotoluene (2,4‐DNT) represents a substantial global health risk, demanding urgent intervention for the removal of this detrimental compound from affected sites and the promotion of ecological restoration. Conventional methodologies, however, are energy‐intensive, susceptible to secondary pollution, and may inadvertently increase carbon emissions. In this study, a 2,4‐DNT degradation module is designed, assembled, and validated in rice plants. Consequently, the modified rice plants acquire the ability to counteract the phytotoxicity of 2,4‐DNT. The most significant finding of this study is that these modified rice plants can completely degrade 2,4‐DNT into innocuous substances and subsequently introduce them into the tricarboxylic acid cycle. Further, research reveals that the modified rice plants enable the rapid phytoremediation of 2,4‐DNT‐contaminated soil. This innovative, eco‐friendly phytoremediation approach for dinitrotoluene‐contaminated soil and water demonstrates significant potential across diverse regions, substantially contributing to carbon neutrality and sustainable development objectives by repurposing carbon and energy from organic contaminants.

## Graphical abstract

This study presents a novel approach to developing environmentally friendly “dinitrotoluene scavengers” plants capable of efficiently degrading 2,4‐dinitrotoluene (2,4‐DNT) into harmless compounds while recovering ATP and acetyl‐CoA through the 2,4‐DNT degradative products. These plants also exhibit a remarkable ability to rapidly phytoremediate 2,4‐DNT, thereby facilitating carbon emission reductions and promoting sustainable development.

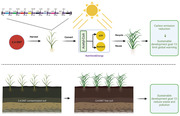

## 1 Introduction

Throughout history, wars and armed conflicts have produced and released billions of tons of trinitrotoluene (TNT) and its hazardous precursor, 2,4‐dinitrotoluene (2,4‐DNT), into the environment, causing significant concern.^[^ ^1^, ^2^ ^]^ 2,4‐DNT is widely used in the production of explosives, as well as in the manufacture of herbicides, plastics, and automobile airbags, resulting in significant environmental pollution and carbon emissions, posing a severe threat to public health.^[^ ^3^, ^4^, ^5^ ^]^ Many published studies have established the carcinogenic effects of 2,4‐DNT on humans^[^ ^6^ ^]^ and mutagenic toxicity to microorganisms, algae, fish, and plants.^[^ ^2^, ^7^ ^]^ Recently, the World Health Organization and the United States Environmental Protection Agency classified 2,4‐DNT as a Group 2B human carcinogen and priority pollutant, respectively^[^ ^8^, ^9^ ^]^ Consequently, the increasing concern about 2,4‐DNT pollution has sparked a frenzy to eliminate it from the environment.

Although there are some conventional remediation techniques for eliminating organic pollutants, these methods are energy intensive, prone to secondary contamination, require the sequestration of carbon to be sustainable, and are incompatible with carbon neutral and sustainable development goals. Aside from proficiently utilizing resources like effluent, sediment, and nutrients, harnessing the energy enclosed within the chemical bonds of environmental organic compounds becomes a vital alternative for achieving net‐zero carbon emissions.^[^ ^10^ ^]^ Some literature demonstrates that using organic contaminants from water bodies and soil sources can produce valuable resources or sustainable energy^[^ ^11^, ^12^ ^]^ Therefore, developing upcycled organic waste for valuable material conversion has garnered considerable interest.

Due to the higher costs associated with traditional methods of remediating nitroaromatic explosives‐contaminated sites,^[^ ^13^ ^]^ alternative strategies such as bacterial or plant remediation, also known as bacterial mineralization or phytoremediation, have emerged. For example, phytoremediation has been studied for its ability to remediate 2,4‐DNT^[^ ^14^ ^]^ and TNT,^[^ ^15^, ^16^ ^]^ which serve as intermediate and final products in the synthesis of nitroaromatic explosives, respectively. The microorganisms are primarily responsible for converting toxic materials into less‐toxic or innocuous materials.^[^ ^17^ ^]^ However, phytoremediation, powered by photosynthesis and solar energy, provides an effective and environmentally‐friendly approach of eliminating 2,4‐DNT.^[^ ^18^ ^]^ Current researchs, including demonstrable successes in the bio‐transformation of potent toxins such as Research Department Explosive (RDX) and 4‐nitrobenzaldehyde, strongly indicate the capability of plants to convert organic pollutants into less hazardous derivatives.^[^ ^19^, ^20^ ^]^ Nevertheless, some studies indicate that organic contaminant degradation generates secondary wastes used as inputs for the subsequent recycling process.^[^ ^21^ ^]^ To address this concern, converting metabolic end‐products into non‐toxic by‐products offers a promising solution. In addition to the complete degradation of 2, 4‐DNT, this study aimed to create an engineered plant that can achieve the following three goals: 1) the complete degradation of 2, 4‐DNT into innoxious substance; 2) the conversion of the 2, 4‐DNT degradation products into useful feedstock/value resources for plant growth; and 3) the digestion of 2, 4‐DNT as a “food” and the absorption of energy/nutrition from this “food.” Numerous scholars agree that acetyl‐CoA is critical in plants for synthesizing a wide range of compounds, including amino acids, isoprenoids, phenolics, flavonoids, and alkaloids.^[^ ^22^ ^]^ Additionally, acetyl‐CoA is catabolized in the tricarboxylic acid (TCA) cycle to generate energy.^[^ ^22^ ^]^ The pathways proposed in this study should therefore facilitate the supply of acetyl‐CoA and the production of acetyl‐CoA‐derived molecules.

The fully elucidated bacterial pathway responsible for 2,4‐DNT mineralization cleaves it into pyruvate and propionyl‐CoA.^[^ ^17^ ^]^ Numerous studies have demonstrated that increased pyruvate synthesis leads to higher acetyl‐CoA levels due to the conversion of pyruvate to acetyl‐CoA by the pyruvate dehydrogenase complex^[^ ^23^, ^24^ ^]^ Additionally, propionyl‐CoA can be converted into acetyl‐CoA in plants via the β‐oxidation pathway.^[^ ^25^ ^]^ This pathway is therefore suitable for modification and transformation in plants for 2,4‐DNT biodegradation and utilization. Although large biomass plants such as hyperaccumulators are better suited for phytoremediation, rice seedlings were chosen as the candidate plants for this study because rice seedlings are a well‐studied model system and are easy to transform. A 2,4‐DNT degradation module was designed and tested in rice plants to evaluate their potential for degrading and utilizing 2,4‐DNT.

## 2 Results

### 2.1 Vector Construction and Identification of Modified Rice Plants

**Figure 1:**
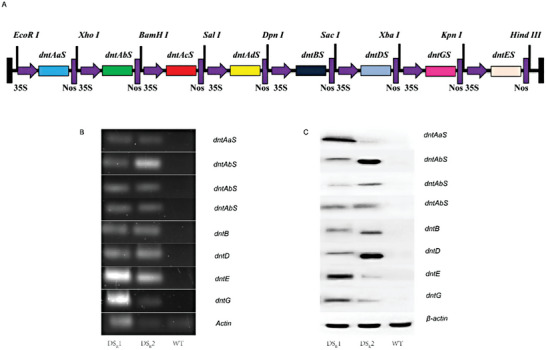
Creation of super “dinitrotoluene scavenger” plants. A) Gene cassette schematic representation of the complete 2,4‐DNT degradation, as designed in this study. *dntAaS*, *dntAbS*, *dntAcS*, and *dntAdS* (encoding four subunits of DNT dioxygenase); *dntBS* (encoding methylnitrocatechol monooxygenase); *dntDS* (encoding trihydroxytoluene oxygenase); *dntGS* (encoding bifunctional isomerase/hydrolase); *dntES* (encoding CoA‐dependent methylmalonate semialdehyde dehydrogenase). B) Real‐time polymerase chain reaction analysis of the expression of genes involved in the synthetic 2,4‐DNT degradative pathway. C) Western blot analysis of the expression of proteins involved in the synthetic 2,4‐DNT degradative pathway. DS_R_1 and DS_R_2 represent two modified rice plants; WT represents wild‐type rice plants.

Previous studies have indicated that *Burkholderia cepacia*, a strain capable of utilizing 2,4‐DNT as its sole carbon, energy, and nitrogen source, possesses the complete degradation pathway for 2,4‐dinitrotoluene, encompassing multiple enzymes including 2,4‐DNT dioxygenase, 4‐methyl‐5‐nitrocathecol (4M5NC) monooxygenase, 2‐hydroxy‐5‐methylquinone (2H5MQ) reductase, 2,4,5‐trihydroxytoluene (2,4,5‐THT) oxygenase, bifunctional isomerase/hydrolase, and CoA‐dependent methylmalonate semialdehyde dehydrogenase.^[^ ^17^, ^26^ ^]^ Among these enzymes, the 2H5MQ reductase (encoded by *dntC*), which catalyzes the conversion of 2H5MQ to 2,4,5‐THT, is a nonspecific reductase.^[^ ^26^ ^]^ To completely degrade 2,4‐DNT in rice plants, a 2,4‐DNT degradative pathway composed of five biodegradative enzymes was reconstructed (Table S1, Supporting Information). Due to 2,4‐DNT dioxygenase being encoded by four genes (*dntAaS*, *dntAbS*, *dntAcS* and *dntAdS*), thus all eight genes encoding 2,4‐DNT degradative route enzymes, namely *dntAaS*, *dntAbS*, *dntAcS*, *dntAdS*, *dntBS*, *dntDS*, *dntGS*, and *dntES* from *Burkholderia cepacia*, were obtained through chemical synthesis according to the bias codons of the plant (Note S1, Supporting Information). The resulting plasmid pYR6535 harbored a 14.5 kb DNA fragment, which contained eight gene expression cassettes in a tandem manner, with every gene surrounded by the CaMV 35S promoter and the NOS terminator (**Figure** **1**A). Thus, the recombinant vector pYR6535 was transformed in 7‐day‐old immature zygotic rice (*Oryza sativa* L. ssp *japonica*, ‘Zhonghua 11′) embryos.

Real‐time polymerase chain reaction analyses were performed to confirm the expression of the eight inserted genes in the dinitrotoluene scavenger rice (DS_R_). As expected, eight exogenous genes (*dntAaS*, *dntAbS*, *dntAcS*, *dntAdS*, *dntBS*, *dntDS*, *dntGS*, and *dntES*) were amplified in dinitrotoluene scavenger rice line 1 and line 2 (DS_R_1 and DS_R_2) seedlings but not in the wild‐type (WT) rice plants (Figure 1B). The proteins generated by the eight 2,4‐DNT degradative route genes were then tested using Western blot analysis to verify whether they were appropriately expressed. In the transgenic lines, the cleaved proteins of *dntAaS*, *dntAbS*, *dntAcS*, *dntAdS*, *dntBS*, *dntDS*, *dntGS*, and *dntES* were detectable, but none of these proteins were detected in WT rice plants (Figure 1C). These results indicated that all inserted foreign genes were successfully expressed and transcribed in dinitrotoluene scavenger rice plants.

### 2.2 Enhancing Resistance to 2,4‐DNT

**Figure 2:**
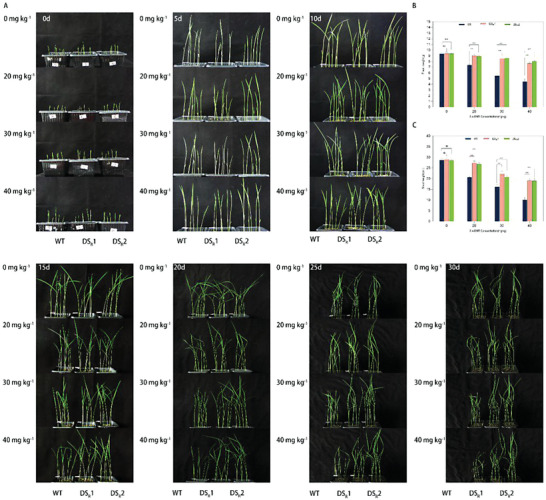
2,4‐DNT resistance assay in the WT and dinitrotoluene scavenger rice seedlings. A) Representative images of the WT and dinitrotoluene scavenger rice seedlings grown in soil supplemented with 2,4‐DNT at concentrations ranging from 0 to 40 mg kg^−1^. B) Comparison of the fresh weight of rice plants grown in medium supplemented with 0, 20, 30, and 40 mg kg^−1^ 2,4‐DNT. C) Shoot length of rice plants grown in medium supplemented with 0, 20, 30, and 40 mg kg^−1^ 2,4‐DNT. Data are presented as the mean ± SD (*n* = 6). NS, no significant difference. *p*‐values were calculated using the Student's *t*‐test. *p < 0.05; **p < 0.01; ***p < 0.001; ****p < 0.0001.

Earlier studies have demonstrated that the phytotoxicity of 2,4‐DNT hinders the germination, biomass, and root development of plants^[^ ^27^, ^28^ ^]^ To verify the 2,4‐DNT resistance of dinitrotoluene scavenger plants, seedlings of the dinitrotoluene scavenger plants and WT plants were exposed to 2,4‐DNT‐containing soil. Compared with the control, the DS_R_1 and DS_R_2 rice lines consistently demonstrated superior growth (**Figure** **2**A). After 30 days of growth in soil containing 2,4‐DNT (0, 20, 30, and 40 mg kg^−1^), the fresh weight of the DS_R_1 and DS_R_2 rice lines was significantly greater than that of the WT plants (*p* < 0.05) (Figure 2B). Similarly, the shoot lengths of the DS_R_1 and DS_R_2 rice lines were significantly increased compared with the WT plants (*p* < 0.05) (Figure 2C). Considering all of this evidence, it seems that the dinitrotoluene scavenger rice plant possesses remarkable 2,4‐DNT resistance capabilities.

### 2.3 2,4‐DNT Uptake and Degradation

**Figure 3:**
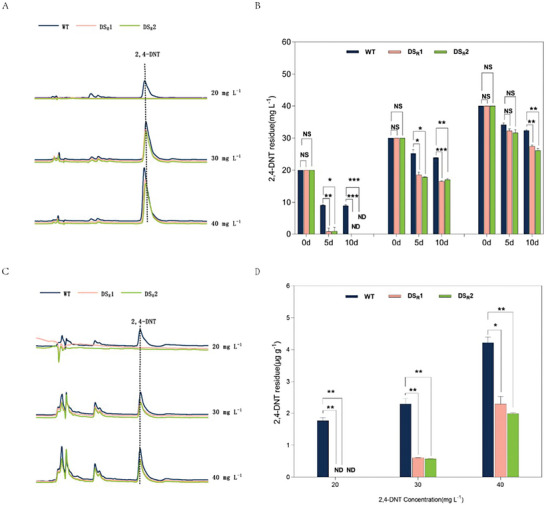
Residual and accumulated 2,4‐DNT in the medium and plants. A) High‐performance liquid chromatography (HPLC) analysis of the residual 2,4‐DNT in medium‐cultivated wild‐type (WT) and dinitrotoluene scavenger rice plants. B) The fate of 2,4‐DNT in the medium‐cultivated WT and dinitrotoluene scavenger rice plants. C) HPLC analysis of accumulated 2,4‐DNT in the WT and dinitrotoluene scavenger rice plants. D) Comparison of 2,4‐DNT accumulation between the WT and dinitrotoluene scavenger rice plants. The WT and dinitrotoluene scavenger rice seedlings grown on medium containing 0, 20, 30, and 40 mg L^−1^ 2,4‐DNT for 10 d. The asterisk at the top of the bar indicates different significances according to Student's *t*‐test. Error bars, mean ± SD (*n* = 2), *p < 0.05; **p < 0.01; ***p < 0.001.

**Figure** **3**A shows the uptake of 2,4‐DNT from the medium by the WT and DS_R_ plants (Figure 3A; Figure S1, Supporting Information). Though the current study is based on a small sample size, our findings suggest that the DS_R_ plant is quicker at taking up 2,4‐DNT compared with the WT plant. The most striking result to emerge from the data is that no 2,4‐DNT could be detected in the medium in which the DS_R_ plant was cultivated, while 8.60 mg L^−1^ 2,4‐DNT remained in the medium in which WT rice plants were cultivated (Figure 3B). This observation may support the hypothesis that the dinitrotoluene scavenger rice plants enormously enhance 2,4‐DNT uptake. Figure 3C shows the accumulation of 2,4‐DNT in the WT and dinitrotoluene scavenger plants after 10 days of treatment (Figure 3C; Figure S2, Supporting Information). The most surprising aspect of the data is that no 2,4‐DNT accumulation was detected in the dinitrotoluene scavenger rice plants, while the 2,4‐DNT concentration in the WT plants reached 1.84 µg g^−1^ (Figure 3D). It is apparent from Figure 3 that the dinitrotoluene scavenger plants took up more but accumulated less 2,4‐DNT than the WT plants. As 2,4‐DNT could not be detected either in the medium or plants at the end of 20 mg L^−1^ 2,4‐DNT treatment, the evidence presented in this section suggests that the dinitrotoluene scavenger plants have the remarkable ability to degrade 2,4‐DNT completely.

### 2.4 Monitoring of 2,4‐DNT Degradation Intermediates

**Figure 4:**
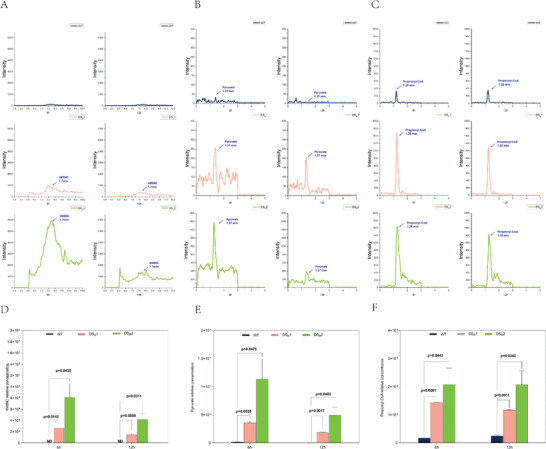
Accumulation of 2,4‐DNT degradation intermediates in plants. A) Gas chromatography‐mass spectrometry (GC‐MS) analysis of the formation of 4M5NC. B) GC‐MS analysis of the formation of pyruvic acid. C) GC‐MS analysis of the formation of propionyl‐CoA. D–F) Relative concentration of 4M5NC, pyruvate, and propionyl‐CoA in the WT and dinitrotoluene scavenger rice plants. The asterisk at the top of the bar indicates different significances according to Student's *t*‐test. Error bars, mean ± SD (*n* = 2), *p < 0.05; **p < 0.01.

To ensure that 2,4‐DNT could be completely degraded by the dinitrotoluene scavenger plants, the presence of 2,4‐DNT and its degradation intermediates methyl nitrocatechol (4M5NC), pyruvate, and propionyl‐CoA was monitored within the plant. The results, presented in **Figure** **4** and Figure S3 (Supporting Information), indicate that 4M5NC was detected in the DS_R_1 and DS_R_2 plants but not in the WT plants, suggesting the correct assembly and enzyme activity of *dntAaAbAcAd* (Figure 4A,D; Figure S3, Supporting Information). Furthermore, the final degradative products pyruvate and propionyl‐CoA were monitored in both the WT and dinitrotoluene scavenger rice plants. Many previous studies indicate that pyruvate and propionyl‐CoA exist in all organisms because they are essential metabolic intermediates in catalyzing the degradation of propionate, odd‐chain fatty acids, and the methylaspartate cycle^[^ ^29^, ^30^ ^]^ The most striking result to emerge from the data is that the relative concentrations of pyruvate and propionyl‐CoA in the dinitrotoluene scavenger plants were higher than those of the WT plants after 6 h of treatment (Figure 4B,C,E,F; Figures S4 and S5, Supporting Information). According to existing knowledge, pyruvate and propionyl‐CoA will enter the tricarboxylic acid cycle and ultimately be metabolized by plants^[^ ^31^, ^32^ ^]^ These striking results suggest that the enzymes encoded by *dntB*, *dntD*, *dntE*, and *dntG* are functional and that all enzymes within the 2,4‐DNT degradative route are correctly assembled, ultimately leading to the metabolism of pyruvate and propionyl‐CoA within the plants (Figure 4). To the best of our knowledge, this is the first reported instance of the complete degradation of 2,4‐DNT into nontoxic metabolites utilizing dinitrotoluene scavenger plants.

**Figure 5:**
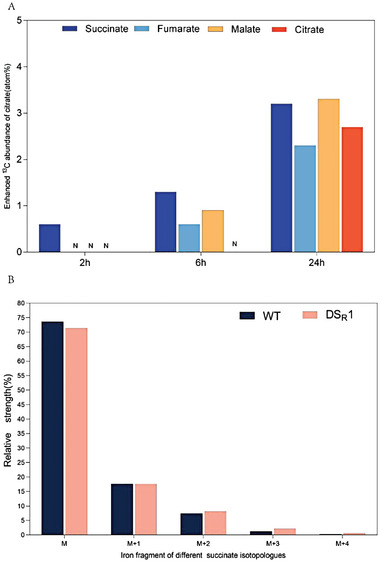
Mass isotopomer analysis of 2,4‐DNT degradation intermediates. A) Mass isotopomer analysis of citrate, succinate, and fumarate in DS_R_1 line by GC‐MS. B) Calculation of mass isotopomer distribution of different succinate isotopologues by uniformization.

Pyruvate and propionyl‐CoA are the end‐products resulting from the breakdown of 2,4‐DNT and are presumed to be transformed into acetyl‐CoA and introduced into the TCA cycle. To validate this assumption, a sextuple 13C‐labeled 2,4‐DNT was used as a tracer. When the dinitrotoluene scavenger plants were fed with external 13C‐labeled 2,4‐DNT, there was a significant increase in the production of 13C‐labeled citrate, succinate, and fumarate (**Figure** **5**A; Table S2, Supporting Information). Additionally, we identified the isotopologues of citrate, succinate, and fumarate, which are critical components of the TCA cycle. Background subtraction revealed a 0.8%, 1.0%, and 0.4% rate for m+2, m+3, and m+4 succinate isotopologues, respectively (Figure 5B). These compelling findings confirm that the dinitrotoluene scavenger plant can efficiently degrade 2,4‐DNT into its TCA cycle and recover “green” energy molecular Adenosine triphosphate (ATP) from 2,4‐DNT degradative products.

### 2.5 Rapid Phytoremediation of 2,4‐DNT‐Contaminated Soil

As the 2,4‐DNT can be converted into acetyl‐CoA and upcycled as “green energy” ATP or feedstock, the dinitrotoluene scavenger plant provides extra energy and feedstock to promote the phytoremediation of 2,4‐DNT‐contaminated soil. To analyze the effectiveness of the phytoremediation of 2,4‐DNT by the WT and dinitrotoluene scavenger plants, the concentrations of the residual 2,4‐DNT in the soil were compared (Figure 7A; Figure S6, Supporting Information). The results showed that the DS_R_1 and DS_R_2 rice plants had no residual 2,4‐DNT at the end of the study, while 41.85 mg kg^−1^ remained in the soil cultivated with the WT rice plants (Figure 7B). These findings highlight the significant benefits of dinitrotoluene scavenger rice plants in enhancing the phytoremediation of 2,4‐DNT‐contaminated soil.

### 2.6 Rhizosphere Bacterial Community Composition Studies

Figure 8 presents a comparative analysis of the rhizosphere bacteria community composition among field‐grown rice plants, comprising WT, DS_R_1, and DS_R_2. A total of 43 genera were identified across all soil samples, with *Massilia, Burkholderia‐Caballeronia‐Paraburkholderia*, and norank‐o‐Chloroplast making up the dominant genera (>5% of total effective sequences). The genera norank‐f‐Micropepsaceae, g‐norank‐f‐Mitochondria, *Phenylobacterium, Allorhizobium‐Neorhizobium‐Pararhizobium‐Rhizobium, Bacillus, Paenibacillus, Sphingomonas*, and norank‐f‐norank‐o‐Saccharimonadales et al. were classified as rare genera (Figure 8A; Table S3, Supporting Information). When comparing the community composition of the dominant and rare genera in the DS_R_1‐ and DS_R_2‐cultivated soil samples with those of WT‐cultivated soil (Figure 8B,C; Tables S4 and S5, Supporting Information) and uncontaminated soil (control) samples (Figure S7; Tables S6–S8, Supporting Information), no significant differences were observed (*P* > 0.05).

## 3 Discussion

Humans engage in various activities that generate anthropogenic waste and emit significant amounts of carbon into the environment. For example, industrial production of 2,4‐DNT necessitates the use of fossil fuels, electricity, and chemical intermediates, thus contributing to the approximated 2 billion metric tons of CO_2_ – constituting ≈5% of global GHGs – emitted annually by the chemical industry.^[^ ^33^ ^]^ Apart from its impact on GHGs emissions, leakage of 2,4‐DNT into the environment can lead to environmental contamination which has become a public concern due to its extensive toxicity and carcinogenic effects. Despite the widespread use of synthetic biology and metabolic engineering in numerous studies aimed at developing plants capable of effectively decomposing contaminants,^[^ ^19^ ^]^ there appear no endeavors in engineering plants specifically tailored for the complete degradation of 2,4‐DNT. Many studies have concluded that sustainable development requires that the problem of organic contamination be addressed by repurposing residual and refuse streams as valuable raw materials.^[^ ^34^ ^]^ Thus, the main objective of this study was to create a plant that can simultaneously consume and utilize 2,4‐DNT. Nevertheless, creating molecular circuits capable of programming complex cellular functions is a fundamental challenge in metabolic engineering applications because the coordinated multigene expression in plants is demanding and strenuous.^[^ ^35^ ^]^

To achieve unimpeded metabolic flow, modifying complex metabolic pathways in plants often requires the simultaneous expression of multiple target genes, or even the entire set of genes within a given pathway.^[^ ^36^ ^]^ Within the spectrum of multiple gene integration strategies, the application of multigene vector transformation, specifically tailored for Agrobacterium‐mediated transformation, emerges with unique benefits: this approach enables integration and inheritance of numerous transgenes within the T‐DNA regions of binary vectors as a unified entity, outpacing alternative methodologies.^[^ ^36^ ^]^ Despite numerous attempts to assemble and transfer complex constructs containing multiple genes, the transfer of large, intact DNA fragments through binary vectors poses a considerable challenge due to their inherent instability and predisposition to spontaneous deletions^[^ ^37^, ^38^ ^]^ It is well‐established through extensive research that the 2,4‐DNT dioxygenase enzyme comprises four unique subunits harmoniously balanced in a 1:1:1:1 ratio,^[^ ^17^ ^]^ implicating the need for the precise engagement of four specific genes (*dntAa*, *dntAb*, *dntAc*, and *dntAd*) for efficient encoding. Thus, a total of eight genes are needed to construct the 2,4‐DNT degradative circuits, which presents significant obstacles to its practical application in multigene vector construction. Previous research has established that genetically modified plants (GMPs) containing numerous transgenes under the control of the same promoter, like the rice plants expressing three betanin biosynthesis genes regulated by the rice globulin‐1 promoter, exhibit coordinated expression.^[^ ^39^ ^]^ It is possible to employ the same promoter for each gene when the coordinated expression is necessary. Therefore, the 35S promoter and terminator were employed to control the gene‐expression process, resulting in the generation of 2,4‐DNT degradative circuits. Benefiting from codon optimization and vector construction, modified rice plants that coordinate the expression of eight 2,4‐DNT degradative genes were obtained (Figure 1).

Recent evidence indicates that plants can uptake DNT through their roots and transport 2,4‐DNT from the roots to the shoots via the xylem.^[^ ^27^ ^]^ Earlier studies have successfully outlined the detoxification process of 2,4‐DNT and nitroaromatic explosives in plants, characterizing it into three distinct stages: first, functionalization, which involves processes such as hydrolysis, oxidation, and reduction; second, transformation, which entails conjugation via glycosyl transferases or glutathione transferases; and lastly, compartmentalization which encompasses transportation toward the cell wall or vacuole.^[^ ^40^ ^]^ Despite these developments, the precise molecular mechanism of the detoxification of nitroaromatic compounds in plants is yet to be wholly understood.^[^ ^40^ ^]^ Recent advancements in bioengineering have shown promising results in the detoxification of dinitrotoluene (DNT) by genetically modifying plant organisms with cyanobacterial flavodoxin and bacterial nitroreductase genes.^[^ ^41^ ^]^ Established literature also confirms that plants acquire xenobiotic tolerance via the integration of a pathway that either degrades or detoxifies the xenobiotics.^[^ ^42^ ^]^ Given that the five enzymes employed to construct the 2,4‐DNTdegradative module in this study serve as detoxifying enzymes in bacteria,^[^ ^17^ ^]^ it is not unusual that these dinitrotoluene scavenger plants displayed resistance to 2,4‐DNT (Figure 2), and exhibited complete 2,4‐DNT degradation capability (Figure 3).

The most crucial finding in this study is that the dinitrotoluene scavenger plant could transform 2,4‐DNT into pyruvate and propionyl‐CoA (Figure 4B,C,E,F). Many published studies have established that pyruvate and propionyl‐CoA can be converted into acetyl‐CoA in plants via the pyruvate dehydrogenase complex (PDC) and β‐oxidation pathway, respectively^[^ ^25^, ^43^, ^44^ ^]^ Previous research has confirmed that increased acetyl‐CoA production is favorable for creating important acetyl‐CoA‐derived chemicals.^[^ ^45^, ^46^ ^]^ As a precursor, acetyl‐CoA is utilized to biosynthesize valuable products such as isoprenoids, terpenoids, fatty acids, and fatty acid‐derived chemicals, flavonoids, stilbenoids, and polyketides.^[^ ^47^ ^]^ Due to the enhanced acetyl‐CoA supply in the modified rice plants, beyond the complete degradation of 2,4‐DNT, our study provides a plant material that is “an alternative organic contaminant to a feedstock upcycling platform”.

**Figure 6:**
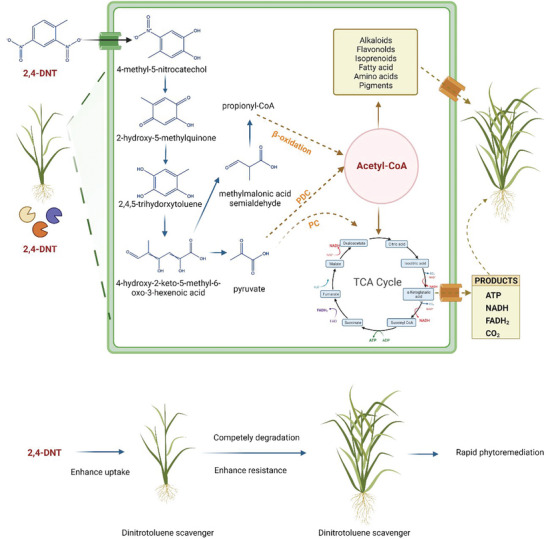
Schematics diagrams illustrating the mechanism of dinitrotoluene scavenger rice plants degrade 2,4‐DNT into an innoxious substance, upcycling of the 2,4‐DNT pollutant to a “green” feedstock and progress of rapid phytoremediation of 2,4‐DNT contamination. PDC, pyruvate dehydrogenase complex; PC, pyruvate carboxylase.

Acetyl‐CoA also can enter the TCA cycle, producing various even‐chain products and other valuable metabolites.^[^ ^24^ ^]^ To date, it has conclusively been shown that increased pyruvate production is conducive to acetyl‐CoA biosynthesis and boosts the TCA cycle level.^[^ ^48^ ^]^ Moreover, pyruvate can enter the TCA cycle directly via the enzyme pyruvate carboxylase (EC 6.4.1.1).^[^ ^24^ ^]^ In the present study, our results indicate that 13C‐labeled 2,4‐DNT can be degraded completely by the dinitrotoluene scavenger rice plant and generate 13C‐labeled citrate, succinate, and fumarate (Figure 5A). This result demonstrates that the 2,4‐DNT pollutant was transformed into acetyl‐CoA and subsequently utilized as a feedstock material to generate ATP in the TCA cycle of the dinitrotoluene scavenger plants (**Figure** **6**). Notwithstanding the relatively limited sample, this work offers valuable insights into organic contaminants‐to‐feedstock research. To the best of our knowledge, the present study is the first to successfully harvest carbon from 2,4‐DNT, convert it into nutrition/energy, and recycle it in plants. In addition, this study holds tremendous potential for promoting the United Nations Sustainable Development Goals (SDGs), implementing carbon‐neutral policies, and fostering a circular economy.

**Figure 7:**
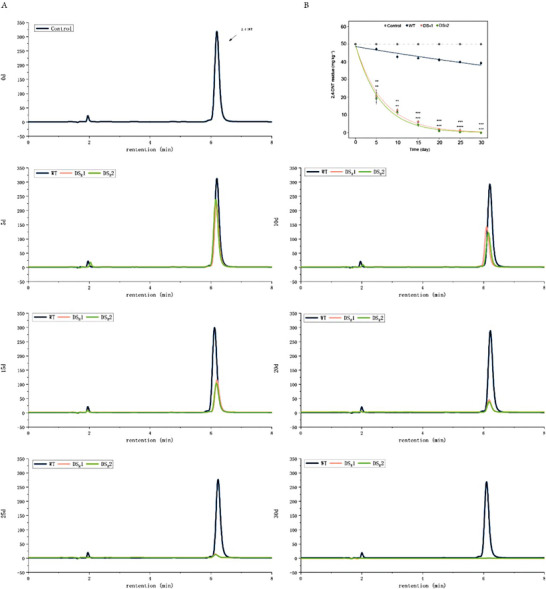
Phytoremediation of 2,4‐DNT‐contaminated soil. A) High‐performance liquid chromatography analysis of the residual 2,4‐DNT in soil cultivated with the WT and dinitrotoluene scavenger rice plants. B) The fate of 2,4‐DNT in soil cultivated with the WT and dinitrotoluene scavenger rice plants. The asterisk at the top of the bar indicates different significances according to Student's *t*‐test. Error bars, mean ± SD (*n* = 2), *p < 0.05; **p < 0.01; ***p < 0.001; ****p < 0.0001.

Phytoremediation has emerged as a viable approach for eliminating hazardous substances and improving soil quality. This method presents a valuable opportunity to reduce our reliance on Earth's resources. Moreover, phytoremediation is often less energy‐intensive when compared with physical and chemical methods. These benefits of phytoremediation highlight its potential as a promising solution for sustainable agriculture and a circular economy.^[^ ^50^ ^]^ It has been demonstrated that the performance of the phytoremediation of contaminats depends on various environmental factors, including plant physiology, plant growth, biomass, and root penetration depth.^[^ ^49^ ^]^ Dinitrotoluene scavenger plants exhibit a notable resistance to 2,4‐DNT phytotoxicity, with an innate capability to completely degrade 2,4‐DNT and accrue extra energy and raw materials to counter 2,4‐DNT toxicity, making them distinctively suitable for the phytoremediation of 2,4‐DNT‐contaminated soil. Consistent with our assumptions, the dinitrotoluene scavenger plant displayed the ability to rapidly phytoremediate 2,4‐DNT‐contaminated soil (**Figure** **7**). The findings of this research provide insights into the in situ phytoremediation of 2,4‐DNT‐contaminated sites.

**Figure 8:**
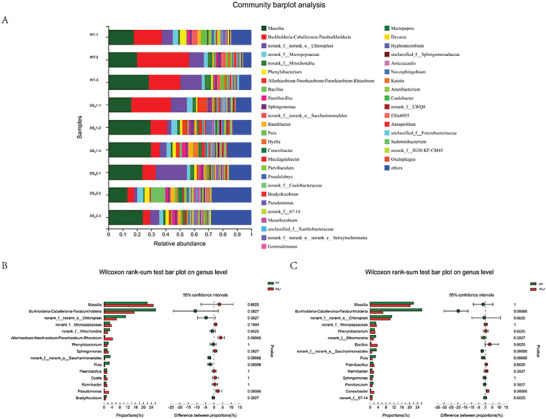
Bacterial community composition in soil cultivated with the WT and dinitrotoluene scavenger rice seedlings. A) The abundance percentages of the bacterial genera in the soil samples. B) The relative abundance of the genera among soil samples cultivated with the WT and DS_R_1 rice seedlings. C) The relative abundance of the genera among soil samples cultivated with the WT and DS_R_2 rice seedlings.

Plants secrete primary and secondary metabolites in their root exudates, which serve as a source of energy for the microbial population in the rhizosphere.^[^ ^51^ ^]^ Additionally, developing plants secrete a variety of metabolites that function as signaling molecules in root exudates to interact with rhizosphere microorganisms^[^ ^52^, ^53^ ^]^ The production of specific proteins from GMPs may change the composition of root metabolites and affect rhizosphere microorganisms.^[^ ^54^ ^]^ A variety of variables might likely have an effect on the composition of the microbial communities in the rhizosphere. As the monitoring of all elements of a soil ecosystem for their reaction to a GMP is impractical, keystone indicators such as the rhizosphere bacterial composition are recommended.^[^ ^55^ ^]^ In the present study, the rhizosphere bacterial community composition of the dominant and rare genera at the genus level did not significantly differ between the WT/ DS_R_1 and WT/ DS_R_2 rice seedlings, respectively (**Figure** **8**B,C). A possible explanation for this might be that this dinitrotoluene scavenger rice plant completely degrades 2,4‐DNT into the substrate of the TCA cycle instead of degrading it to fewer toxic substances and secreting it in vitro. This property significantly reduces the effect of dinitrotoluene scavenger rice plants on rhizosphere bacteria. On a preliminary basis, the available data suggest that these dinitrotoluene scavenger plants exert a negligible effect on rhizosphere microbial community. A further study should assess the long‐term interaction effects between the rhizosphere microbial community and 2,4‐DNT scavenger plants.

## 4 Conclusion

In summary, this study presents novel dinitrotoluene scavenger plants that can completely biodegrade 2,4‐DNT into an innoxious substance. Besides this, these plants not only recover ATP and acetyl‐CoA through 2,4‐DNT metabolic conversion, but also expedite the phytoremediation of 2,4‐DNT‐contaminated soils. These findings offer valuable insights into the potential for sustainable phytoremediation through the use of environmentally friendly upcycling plants at 2,4‐DNT‐contaminated sites, thereby promoting carbon neutrality and sustainable development.

## 5 Experimental Section

### Degradation Route Design and Vector Construction

To construct the gene expression cassette, eight 2,4‐DNT degradative pathway genes, namely *DntAa, DntAb, DntAc, DntAd* (encoding the four subunits of 2,4‐DNT dioxygenase)*, DntB* (encoding methylnitrocatechol monooxygenase), *DntD* (encoding trihydroxytoluene oxygenase)*, DntE* (encoding CoA‐dependant methylmalonate semialdehyde dehydrogenase), *and DntG* (encoding bifunctional isomerase/ hydrolase) from *Burkholderia cepacia*, were chemically synthesized according to the bias codons of plant via the PCR‐based two‐step DNA synthesis method.^[^ ^56^ ^]^ Synthetic *DntAa, DntAb, DntAc, DntAd, DntB, DntD, DntE*, and *DntG* were named *DntAaS, DntAbS, DntAcS, DntAdS, DntBS, DntDS, DntES*, and *DntGS* (The sequence information can be found in Table S1 and Note S1, Supporting Information). All synthesized genes were seamlessly connected with CaMV 35S/TMV omega (Genbank: AY183361) and terminator to construct gene expression cassette via the modified overlap‐extension PCR method.^[^ ^57^ ^]^ The PCR products were purified, cloned into the pGEM‐T easy vector, and sequenced before cloning in the expression vector. At last, the gene expression cassettes for *dntAaS*, *dntAbS*, *dntAcS*, *dntAdS*, *dntBS*, *dntDS*, *dntES*, and *dntGS* were digested with *EcoR I*, *XhoI*, *BamH I*, *Sal I*, *Dpn I*, *Sac I*, *XbaI*, *Kpn I*, and *Hind III*, then inserted into a modified pCAMBIA1301 vector step by step, yielding the final constructs, pYR6535 (Figure. 2).

### Plant Transformation and RT‐PCR Analysis of Integrated Genes

The final constructs, pYR6535, were introduced into *Agrobacterium tumefaciens* strain EHA105 by electroporation. Callus induction and Agrobacterium‐mediated genetic transformation were performed according to the method of Hiei and Komari.^[^ ^58^ ^]^ The homozygous genotypes of modified plants were obtained from self‐fertilization and confirmed by the RT‐PCR. Semi‐quantitative PCR reactions were conducted to confirm the transcription of all genes and evaluate their relative expression. A pair of oligonucleotide primers (RAc1Z1:5′‐ 5′‐AAGATCCTGACGGAGCGTGGTTAC‐3′; RAc1F1: 5′‐ CTTCCTAATATCCACGTCGCACTTC‐3′) specific to rice actin gene RAc1 (GenBank X16280) were used as the positive internal control for PCR analyses. The primer sequences used in this study are listed in Note S2 (Supporting Information).

### Western Blot Analysis

The protein fraction from the rice seedlings was extracted with SDS‐Tris buffer (0.1 m Tris‐HCl, 5% SDS, and 2% β‐mercaptoethanol, pH 6.8) and standardized for protein concentration. Tris/glycine/SDS buffer was used to separate protein samples on a 3% gel and a 10–15% separation gel before transferring them onto a nitrocellulose membrane. Eight distinct proteins were evaluated for specificity using rabbit‐derived specific antibodies (Abclonal Biotechnology Co., China) at a 1:1000 dilution, and goat anti‐rabbit IgG labeled with alkaline phosphatase was used as the secondary antibody. The results were developed and visualized using the substrate electrochemiluminescence technique, and X‐ray imaging was performed.

### Phytotoxic Resistance Assay

To analyze phytotoxicity, growth parameters, such as plant fresh weight and root length, were measured. Six rice seedlings (5 days old) were carefully transferred to soil supplemented with 0, 20, 30, and 40 mg kg^−1^ of 2,4‐DNT. The rice seedlings were placed in a greenhouse at a temperature of 22–25 °C. After 30 days, the plant material was collected and the root length and fresh weight of each rice seedling were measured.

### 2,4‐DNT Uptake Assay

Ten‐day‐old rice seedlings (10 per flask) were aseptically transferred into 200 mL of hydroponic Murashige & Skoog (MS) medium spiked with 2,4‐DNT at concentrations of 0, 20, 30, and 40 mg L^−1^ under a 16‐h light/8‐h dark photoperiod at 25°C. Samples of the hydroponic MS medium were collected at intervals of 0, 5, and 10 days, with two replicates each, to determine the residual 2,4‐DNT. After a 10‐day growth period, the rice plants were harvested, washed with distilled water, ground, and suspended in 1 mL of water. High‐performance liquid chromatography was used to analyze the residual 2,4‐DNT in the rice plants and medium samples to confirm the uptake of all 2,4‐DNT.

### Quantification of 2,4‐DNT Using High‐Performance Liquid Chromatography (HPLC)

Quantification of 2,4‐DNT was performed using an Agilent 2000 HPLC and a C18 column, with detection at a 244 nm absorption wavelength. The mobile phase included a 60:40 mixture of methanol and water, with a flow rate of 0.6 mL min^−1^. Samples were injected (20 µL) into the column and the retention times and spectra of known standards were used to identify 2,4‐DNT. Standards of 2,4‐DNT were used for quantitation (Promochem, Herts, UK).

### Quantification of 2,4‐DNT Degradative Intermediates

To identify the intermediate products of 2,4‐DNT degradation, including 4M5NC, pyruvate, and propionyl‐CoA, plant samples were frozen with liquid nitrogen and then dissolved with 5 mL of ultrapure water. After 1 h of shaking on a shaker, the extract was centrifuged at 10 000 r min^−1^ for 20 min, and the resulting supernatant was subjected to vacuum freeze‐drying (MARTIN CHRIST, 50D‐37520) at −60°C to remove excess water. The resulting dehydrated samples were reconstituted in 100 µL of ultrapure water and transferred into LC vials for ultra‐performance liquid chromatography‐tandem mass spectrometry (UPLC‐MS/MS) analysis using a Thermo Vanquish UPLC system with a C18‐AQ column (4.6 × 250 mm, SHIMADZU) connected to a Thermo TSQ Quantum Mass Spectrometer. The MS was operated in negative ion and selected reaction monitoring (SIM) modes under optimized parameters, including ion spray voltage (3500 V), nebulizer gas (GS1), auxiliary gas (GS2), and curtain gas (CUR). Samples were injected into the mobile phase flow (80% A:20% B; A:0.1 vol% formic acid in water; B: methanol) at a flow rate of 1 mL min^−1^ using an autosampler.

### Phytoremediation of 2,4‐DNT in the Soil

The garden soil (without 2,4‐DNT) was dried using air and then sieved through a 2‐mm mesh to eliminate stones, debris, and roots. Six robust rice seedlings (3 weeks old) were transplanted into soil spiked with 50 mg kg^−1^ of 2,4‐DNT under a 16‐h light/8‐h dark photoperiod at 25 °C. The soil samples, each with three replicates, were collected at various intervals (0, 5, 10, 15, 20, 25 and 30 days) to extract 2,4‐DNT. After a 30‐day treatment, the plants were harvested, washed with tap water followed by distilled water, freeze‐dried, and weighed. HPLC was used to analyze the residual 2,4‐DNT in the soil as stated above.

### Bacterial 16S rRNA Gene Sequencing

The soil designated for the 16S rRNA gene sequencing analysis was prepared according to the protocol outlined in the phytoremediation experiment. Then, the soil was supplemented with 2,4‐DNT to a final concentration of 50 mg kg^−1^. After this supplementation, the soil was set aside for 3 days to enable uniform distribution of 2,4‐DNT. Post this period, six robust rice seedlings (3 weeks old) were transplanted into soil and cultivated under a 16‐h light / 8‐h dark photoperiod at 25 °C. At the end of the 30‐day treatment, 12 independent samples from the soil cultivated rice seedlings were selected for Illumina MiSeq sequencing of 16S rRNA genes. The sequencing was performed by Shanghai Majorbio Bio‐pharm Technology Co., Ltd. and the resulting data were analyzed on the Majorbio Cloud Platform (www.majorbio.com).

### Statistical Analysis

The statistical analyses were performed using GraphPad Prism 9 software. The significance of the comparison between the two groups was assessed using an unpaired two‐tailed Student's *t*‐test. Specific *p*‐values were delineated within the corresponding figures.

## Conflict of Interest

The authors declare no conflict of interest.

## Author Contributions

J.‐J.G., Z.‐J.L., and B.Z. contributed equally to this work and are co‐first authors. J.‐J.G. performed project administration and wrote original draft. Z.‐J.L. performed conceptualization. B.Z. acquired resources. L.‐J.W. performed visualization. J.X. performed data curation. B.W. performed project administration. X.‐Y.F. and H.‐J.H. performed investigation. Y.‐D.D. and W.‐H.Z. performed formal Analysis. Y.W. acquired software. Z.‐H.Z. performed methodology. Y.‐S.T. performed validation. R.‐H.P. wrote review and edited the manuscript. Q.‐H.Y. performed supervision.

## Supporting information

Supporting Information

## Data Availability

The data that support the findings of this study are available in the supplementary material of this article.
