# Supplementary material for: Creation of Environmentally Friendly Super “Dinitrotoluene Scavenger” Plants
Source: Adv Sci (Weinh). 2023 Sep 15;10(30):2303785. doi: 10.1002/advs.202303785 (PMC10602510; doi:10.1002/advs.202303785)
Supplement: Supplementary file 1 — Supporting Information [file ADVS-10-2303785-s001.pdf]

ADVANCED SCIENCE
Open Access

Supporting Information

for *Adv. Sci.*, DOI 10.1002/advs.202303785

Creation of Environmentally Friendly Super "Dinitrotoluene Scavenger" Plants

Jian-Jie Gao, Zhen-Jun Li, Bo Zhu, Li-Juan Wang, Jing Xu, Bo Wang, Xiao-Yan Fu, Hong-Juan Han, Wen-Hui Zhang, Yong-Dong Deng, Yu Wang, Zhi-Hao Zuo, Ri-He Peng, Yong-Sheng Tian* and Quan-Hong Yao**

Creation of environmentally friendly super "dinitrotoluene scavenger" plants

Jian-Jie Gao[1,2,3#], Zhen-Jun Li[1,2,3#] , Bo Zhu[4#], Li-Juan Wang[1,2,3], Jing Xu[1,2,3], Bo Wang[1,2,3] , Xiao-yan Fu [1,2,3], Hong-juan Han[1,2,3], Wen-Hui Zhang[1,2,3], Yong-Dong Deng[1,2,3], Yu Wang[1,2,3] , Zhi-Hao Zuo[1,2,3], Yong-Sheng Tian [1,2,3*], Ri-He Peng[1,2,3*], Quan-Hong Yao [1,2,3*]

1. Biotechnology Research Institute, Shanghai Academy of Agricultural Sciences, Shanghai 201106, China

2. Shanghai Key Laboratory of Agricultural Genetics and Breeding, Shanghai Academy of Agricultural Sciences, Shanghai 201106, China

3. Key Laboratory for Safety Assessment (Environment) of Agricultural Genetically Modified Organisms, Ministry of Agriculture and Rural Affairs, China

4. Key laboratory for the conservation biological resources, An hui province, College of life sciences, Anhui normal university, wuhu 241000, china

#Jian-Jie Gao, Zhen-Jun Li, Bo Zhu contributed equally to this article
*To whom correspondence should be addressed:
Prof. Yong-Sheng Tian
2901 Beidi Road
Shanghai, 201106, China
Biotechnology Research Institute of Shanghai Academy of Agricultural Sciences
Tel: 86-21-62203180
Fax: 86-21-62205704
Email: tys810508@126.com

Prof. Ri-He Peng
2901 Beidi Road
Shanghai, 201106, China
Biotechnology Research Institute of Shanghai Academy of Agricultural Sciences
Tel: 86-21-62203180
Fax: 86-21-62205704
Email: pengrihe@163.com

Prof. Quan-Hong Yao
2901 Beidi Road
Shanghai, 201106, China
Biotechnology Research Institute of Shanghai Academy of Agricultural Sciences
Tel: 86-21-62203180
Fax: 86-21-62205704
Email: yaoquanhong_sh@aliyun.com

Supplementary data

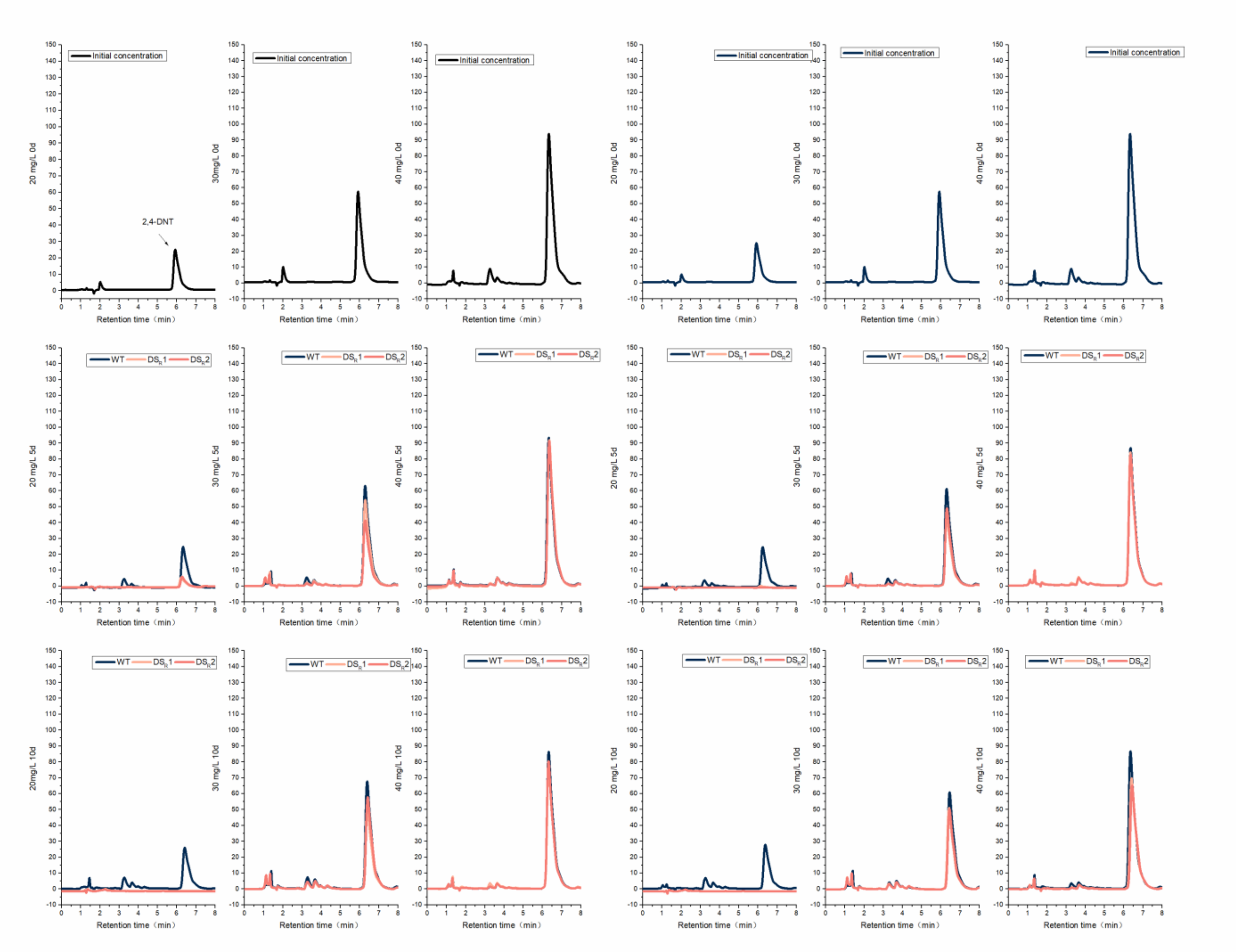

Figure S1. HPLC analysis of residual 2,4-DNT in medium cultivated WT, DS_R1 and DS_R2 rice plants.

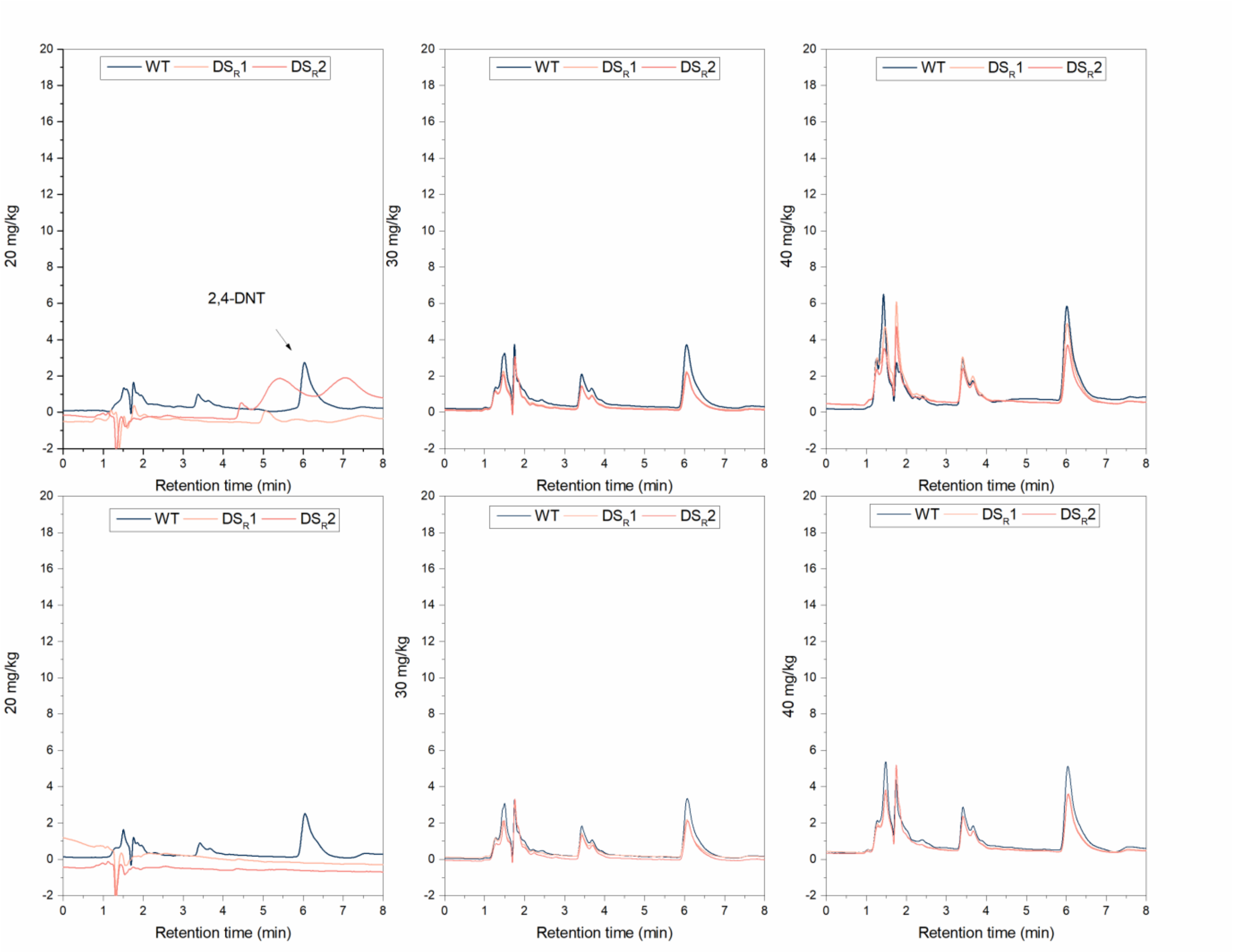

Figure S2. HPLC analysis of 2,4-DNT accumulation between WT and dinitrotoluene scavenger rice plants.

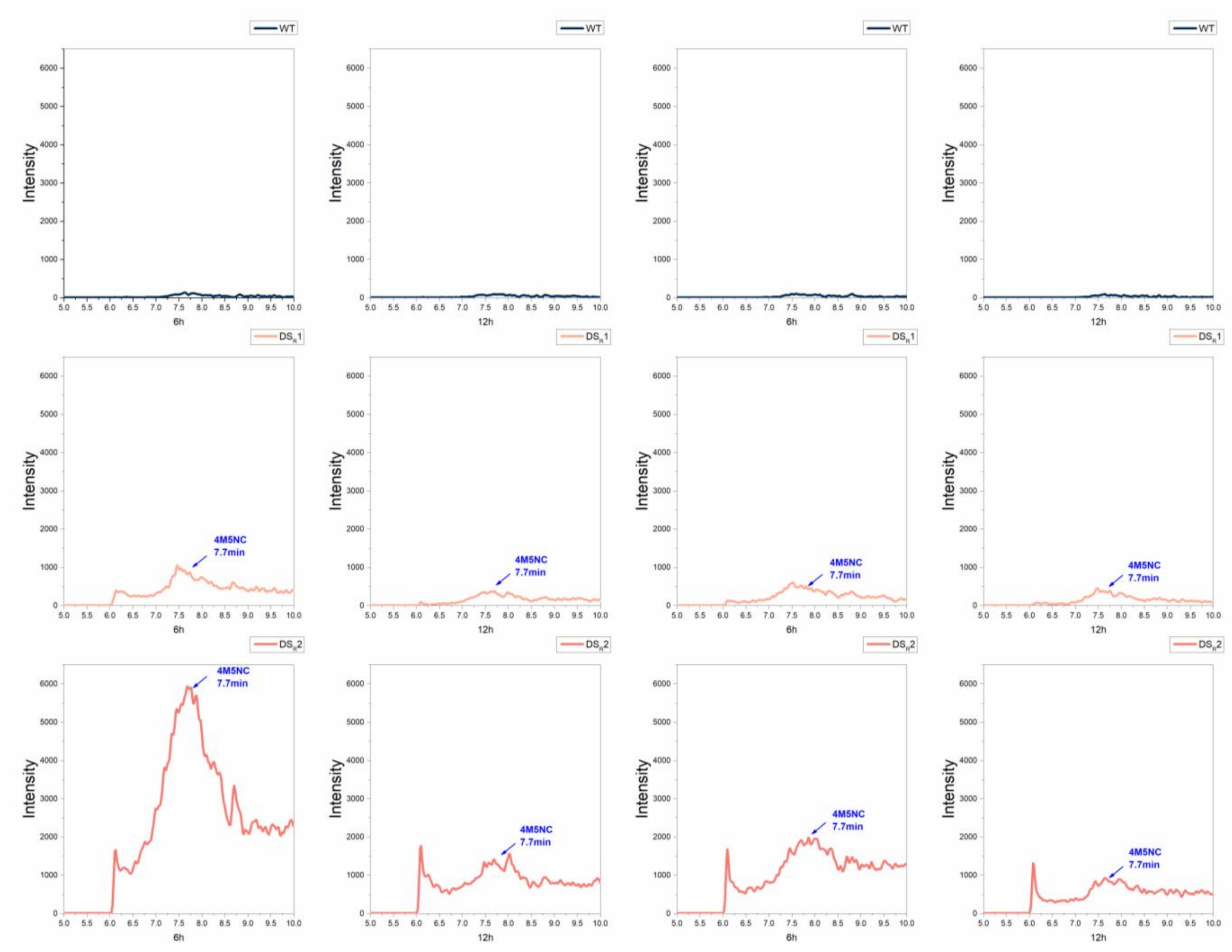

Figure S3. GC-MS analysis of the formation of 4M5NC.

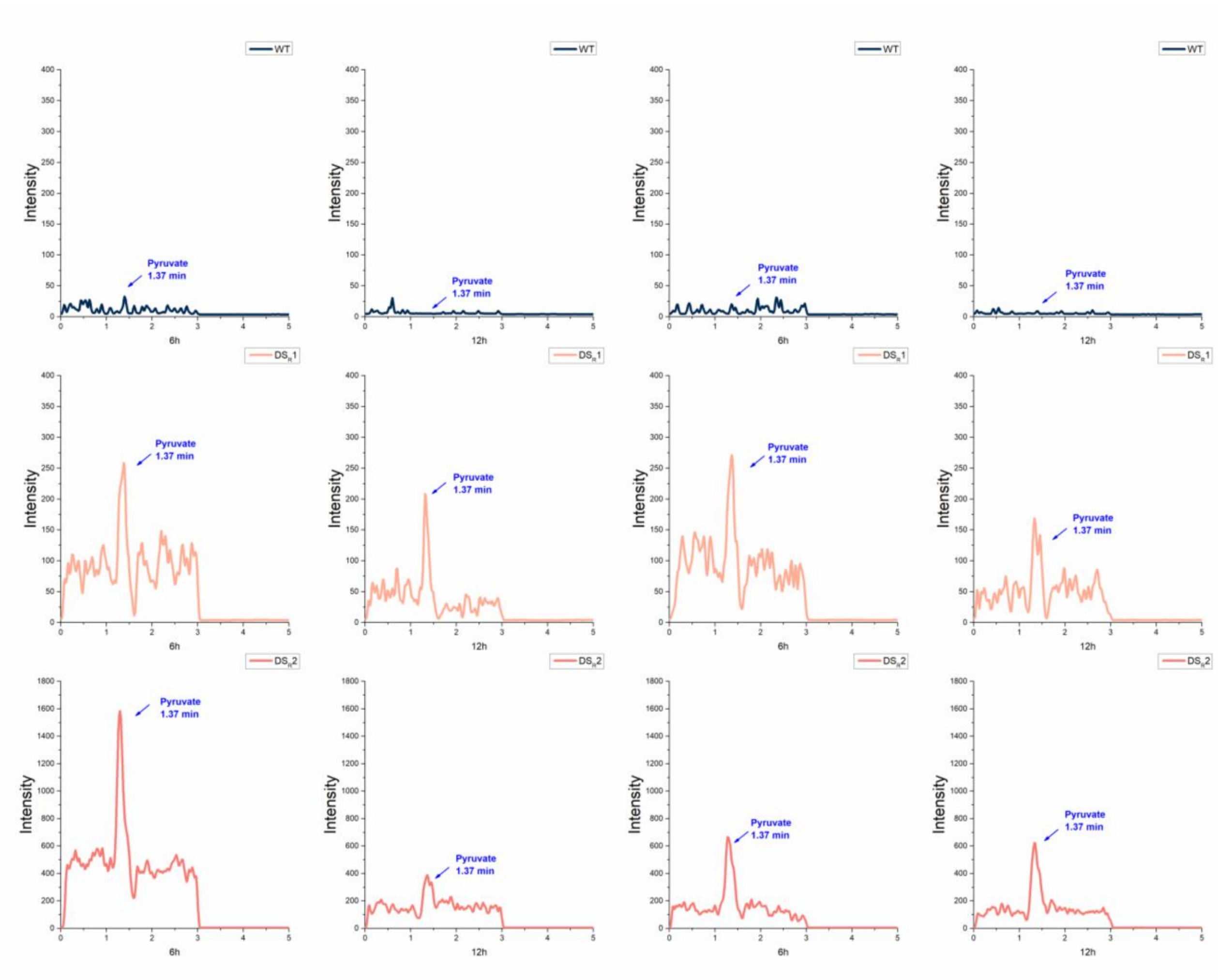

Figure S4. GC-MS analysis of the formation of pyruvate.

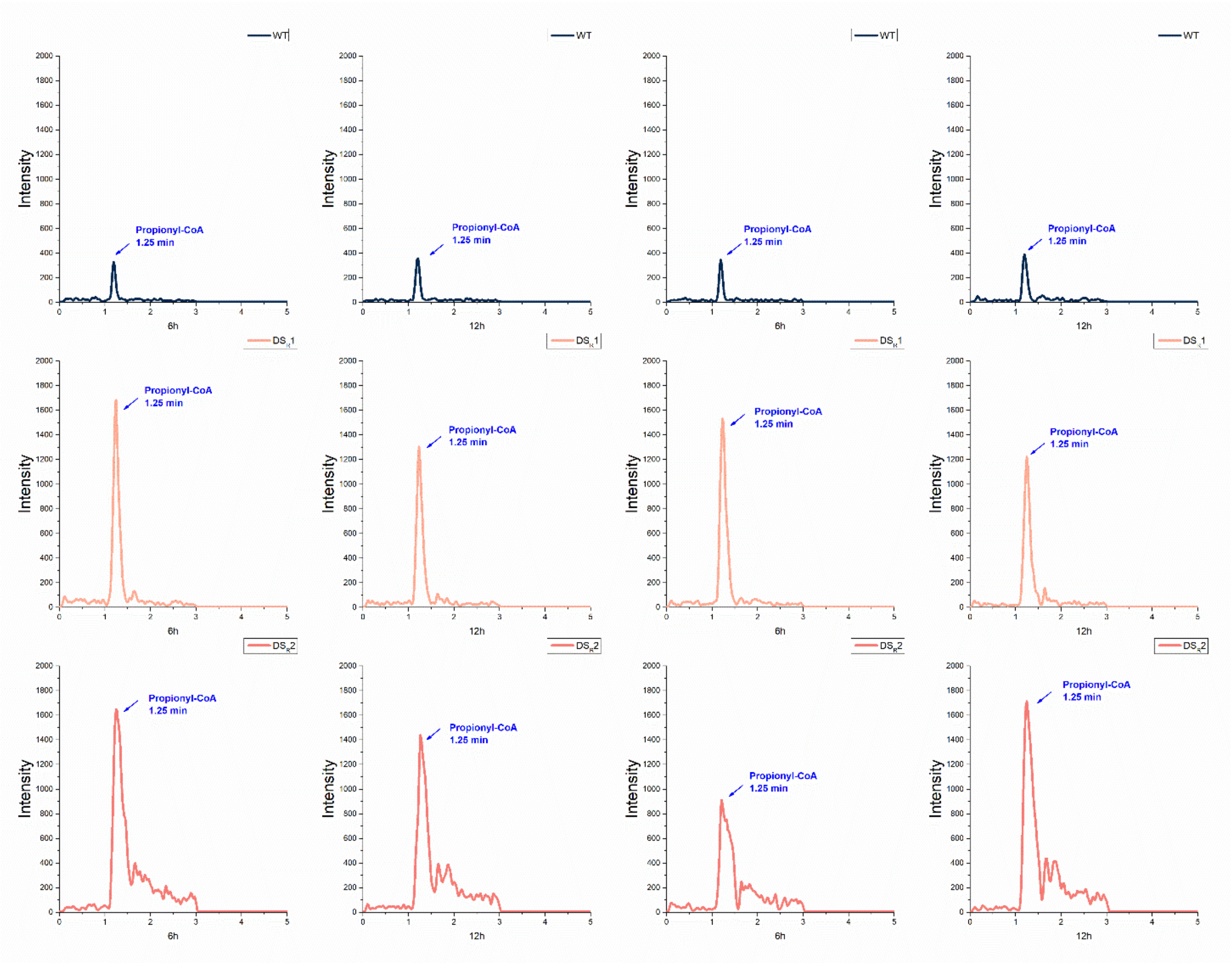

Figure S5. GC-MS analysis of the formation of propionyl-CoA.

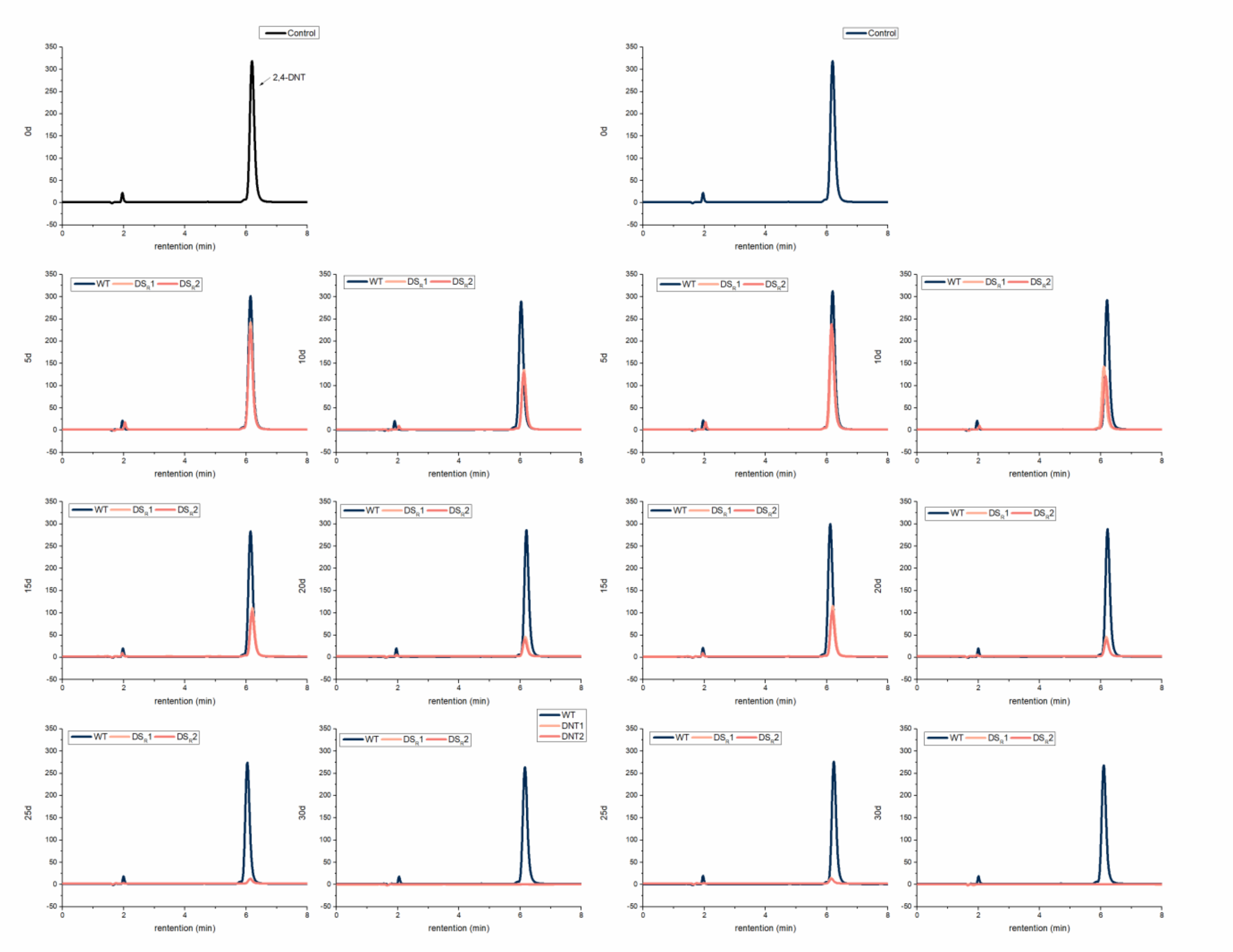

Figure S6. HPLC analysis of residual of 2,4-DNT in soil cultivated WT and dinitrotoluene scavenger rice plant.

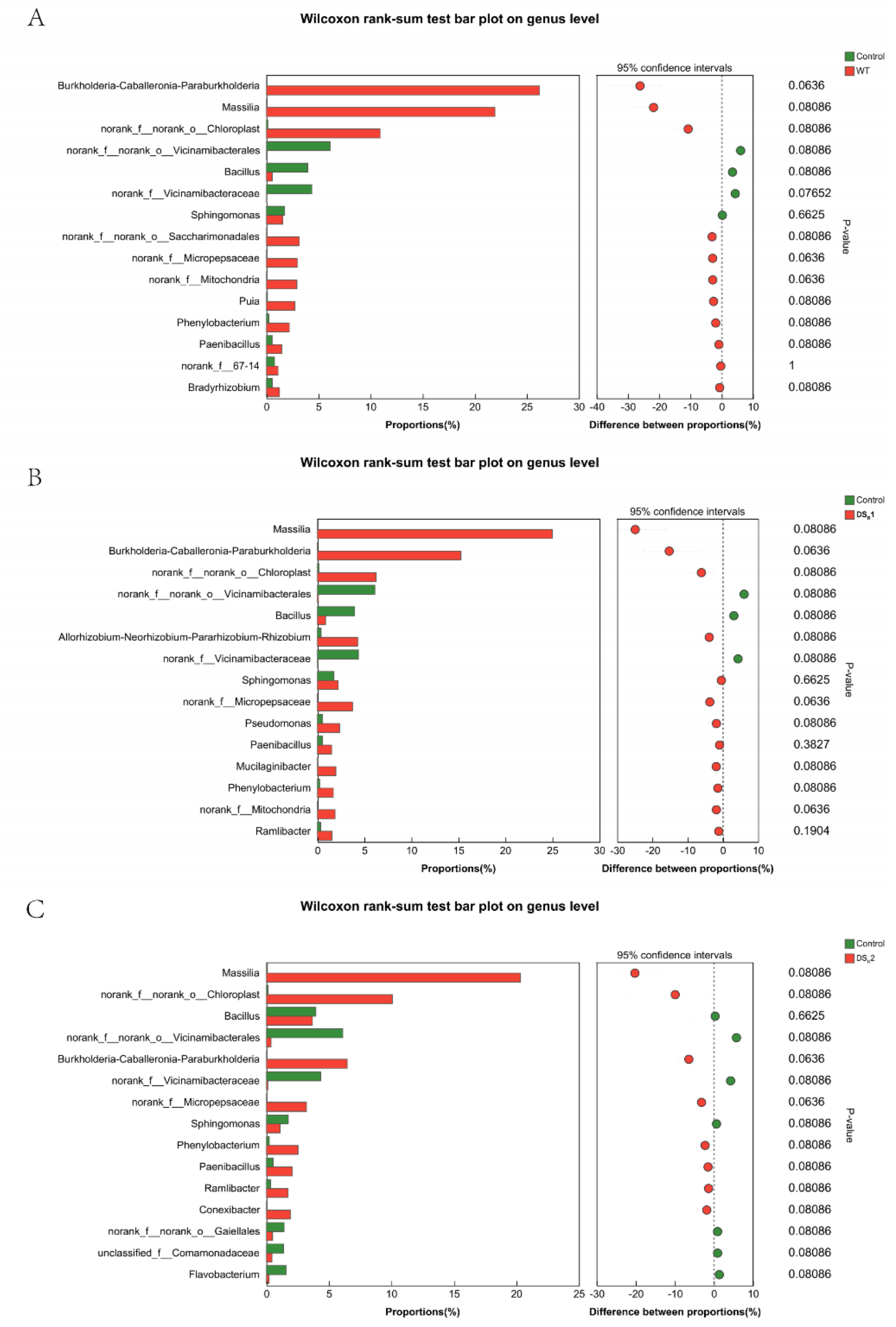

Figure S7. Bacterial community composition in uncontaminated soil (control) and soil cultivated with the WT/ dinitrotoluene scavenger rice seedlings.

Note S1. The sequence information after codon optimization

```
Done on DNA sequence DNTAaS
Total number of bases is: 987.
Analysis done on the complete sequence.
          10        20        30        40        50        60        70
 ATGGAACTTGTTGTTGAACCACTTAATCTTCATCTTAACGCTGAAACTGGTTCTACTCTTCTGGATGTTCTG
  M  E  L  V  V  E  P  L  N  L  H  L  N  A  E  T  G  S  T  L  L  D  V  L
        80        90       100       110       120       130       140
 AGATCCAACGAAGTTCCTATCTCCTACTCTTGTATGTCTGGTAGATGTGGTACTTGCAGATGCAGAGTCATC
  R  S  N  E  V  P  I  S  Y  S  C  M  S  G  R  C  G  T  C  R  C  R  V  I
     150       160       170       180       190       200       210
 GCTGGACACCTGAGAGACAACGGACCTGAGACTGGTAGACCACAGGCTGGTAAGGGTGCTTACGTTCTTGCT
  A  G  H  L  R  D  N  G  P  E  T  G  R  P  Q  A  G  K  G  A  Y  V  L  A
   220       230       240       250       260       270       280
 TGCCAGGCTGTCCTGACCGAAGACTGCACCATCGAGATTCCTGAATCCGACGAGATCGTCGTTCATCCTGCT
  C  Q  A  V  L  T  E  D  C  T  I  E  I  P  E  S  D  E  I  V  V  H  P  A
290       300       310       320       330       340       350       360
 AGAATCGTCAAGGGTACTGTTACTGCTATCGACGAAGCTACTCATGACATCAGAAGACTGAGAATCAAGCTG
  R  I  V  K  G  T  V  T  A  I  D  E  A  T  H  D  I  R  R  L  R  I  K  L
          370       380       390       400       410       420       430
 GCTAAGCCACTGGAGTTCTCTCCTGGACAGTACGCTACTGTCCAGTTCACTCCTGAGTGTGTCAGACCATAC
  A  K  P  L  E  F  S  P  G  Q  Y  A  T  V  Q  F  T  P  E  C  V  R  P  Y
       440       450       460       470       480       490       500
 TCTATGGCTGGTCTGCCATCTGACGCTGAAATGGAGTTCCAAATCAGAGCTGTTCCTGGTGGTCACGTCTCC
  S  M  A  G  L  P  S  D  A  E  M  E  F  Q  I  R  A  V  P  G  G  H  V  S
     510       520       530       540       550       560       570
 AACTACGTCTTCAACGAGCTGTCCGTTGGTGCTTCCGTCAGAATCTCTGGTCCACTTGGTACTGCCTACCTG
  N  Y  V  F  N  E  L  S  V  G  A  S  V  R  I  S  G  P  L  G  T  A  Y  L
   580       590       600       610       620       630       640
 AGAAGAACCCATACTGGTCCTATGCTGTGTGTTGGTGGTGGTACTGGTCTGGCACCTGTCCTGTCCATCGTC
  R  R  T  H  T  G  P  M  L  C  V  G  G  G  T  G  L  A  P  V  L  S  I  V
650       660       670       680       690       700       710       720
 AGAGGTGCACTGGAGTCTGGTATGTCCAACCCTATCCACCTGTACTTCGGTGTCAGATCCGAACAGGACATC
  R  G  A  L  E  S  G  M  S  N  P  I  H  L  Y  F  G  V  R  S  E  Q  D  I
          730       740       750       760       770       780       790
 TACGACGAGGAGAGACTGCACGCACTGGCTGCAAGATTCCCAAACCTGAAGGTCAATGTTGTCGTTGCAACC
  Y  D  E  E  R  L  H  A  L  A  A  R  F  P  N  L  K  V  N  V  V  V  A  T
```

800 810 820 830 840 850 860
GGTCCTGCTGGTCCTGGTCACAGATCCGGTCTGGTCACTGATCTGATCGGTAGAGACCTGCCAAACTTGGCT
G P A G P G H R S G L V T D L I G R D L P N L A
870 880 890 900 910 920 930
GGATGGAGAGCTTACCTGTGTGGTGCTCCTGCTATGGTTGAGGCTCTGAACCTGCTGGTCGCTAGACTGGGT
G W R A Y L C G A P A M V E A L N L L V A R L G
940 950 960 970 980
ATCGTCCCTGGTCACATCCATGCTGACGCATTCTACCCTTCCGGTGTCTAA
I V P G H I H A D A F Y P S G V -

Done on DNA sequence DNTAbS
Total number of bases is: 315.
Analysis done on the complete sequence.
10 20 30 40 50 60 70
ATGTCTGAGAACTGGATTGATGCTGCTGCTAGAGATGAGGTTCCTGAGGGTGATGTCATCGGTATCAACATC
M S E N W I D A A A R D E V P E G D V I G I N I
80 90 100 110 120 130 140
GTTGGCAAGGAGATCGCTCTGTACGAGGTTGCTGGTGAAATCTACGCTACCGACAACACCTGCACTCATGGT
V G K E I A L Y E V A G E I Y A T D N T C T H G
150 160 170 180 190 200 210
GCTGCTAGAATGTCCGATGGTTTCCTGGAAGGTAGAGAGATCGAATGTCCATTGCATCAAGGTAGATTCGAC
A A R M S D G F L E G R E I E C P L H Q G R F D
220 230 240 250 260 270 280
GTCTGTACTGGTAAAGCACTGTGCACTCCACTGACTCAGGACATCAAGACCTACCCTGTCAAGATCGAGAAC
V C T G K A L C T P L T Q D I K T Y P V K I E N
290 300 310
ATGAGAGTCATGCTGAAGCTGGACTAA
M R V M L K L D -

Done on DNA sequence DNTAcS
Total number of bases is: 1344.
Analysis done on the complete sequence.
10 20 30 40 50 60 70
ATGTCTTACCAGAACTTGGTTTCTGAAGCTGGTCTGACTCAGAAGCATCTGATCTATGGTGACAAGGAACTC
M S Y Q N L V S E A G L T Q K H L I Y G D K E L
80 90 100 110 120 130 140
TTCCAGCACGAACTGAAGACCATCTTCGCTAGAAACTGGCTGTTCCTGACCCATGACTCTCTGATTCCATCT
F Q H E L K T I F A R N W L F L T H D S L I P S
150 160 170 180 190 200 210
CCTGGTGACTACGTCAAAGCTAAGATGGGTGTTGACGAAGTCATCGTCTCCAGACAGAACGACGGTTCTGTC
P G D Y V K A K M G V D E V I V S R Q N D G S V
220 230 240 250 260 270 280

AGAGCATTCTTGAACGTTTGCAGACACAGAGGTAAGACCATCGTCGATGCTGAAGCTGGTAACGCTAAGGGT
R A F L N V C R H R G K T I V D A E A G N A K G
290 300 310 320 330 340 350 360
TTCGTCTGTGGTTATCACGGTTGGGGTTACGGTTCCAACGGTGAACTGCAATCCGTCCCATTCGAGAAGGAG
F V C G Y H G W G Y G S N G E L Q S V P F E K E
370 380 390 400 410 420 430
CTGTACGGTGACGCTATCAAGAAGAAGTGCCTGGGTCTGAAGGAAGTTCCTAGAATCGAGTCCTTCCACGGT
L Y G D A I K K K C L G L K E V P R I E S F H G
440 450 460 470 480 490 500
TTCATCTACGGTTGCTTCGACGCTGAAGCACCACCACTGATCGACTACCTGGGTGATGCTGCTTGGTATCTG
F I Y G C F D A E A P P L I D Y L G D A A W Y L
510 520 530 540 550 560 570
GAACCAACCTTCAAGCACTCTGGTGGTCTGGAACTCGTTGGTCCACCTGGTAAGGTCGTCGTCAAGGCTAAC
E P T F K H S G G L E L V G P P G K V V V K A N
580 590 600 610 620 630 640
TGGAAGCCACTTGCTGAGAACTTCGTTGGTGACGTCTACCACATCGGTTGGACCCACGCATCCATCCTGAGA
W K P L A E N F V G D V Y H I G W T H A S I L R
650 660 670 680 690 700 710 720
GCTGGTCAGTCCATCTTCGCTCCACTTGCTGGTAACGCTATGTTCCCACCTGAAGGTGCTGGTCTTCAGATG
A G Q S I F A P L A G N A M F P P E G A G L Q M
730 740 750 760 770 780 790
ACCACCAAGTACGGTTCTGGTATCGGTGTCTTGTGGGATGCTTACTCTGGTATCCAGTCTGCTGACATGGTC
T T K Y G S G I G V L W D A Y S G I Q S A D M V
800 810 820 830 840 850 860
CCTGAGATGATGGCATTCGGTGGTGCTAAGCAGGAGAAGCTGGCTAAGGAGATCGGTGACGTCAGAGCAAGA
P E M M A F G G A K Q E K L A K E I G D V R A R
870 880 890 900 910 920 930
ATCTACAGATCCCAACTGAACGGTACTGTCTTCCCAAACAACTCCTTCTTGACCTGCTCTGGTGTCTTCAAG
I Y R S Q L N G T V F P N N S F L T C S G V F K
940 950 960 970 980 990 1000
GTCTTCAACCCAATCGACGAGAACACCACTGAGGTCTGGACCTACGCTATCGTCGAGAAGGACATGCCTGAG
V F N P I D E N T T E V W T Y A I V E K D M P E
1010 1020 1030 1040 1050 1060 1070 1080
GACTTGAAGAGAAGACTGGCTGATGCTGTTCAGAGATCCGTTGGTCCTGCTGGTTACTGGGAGTCCGATGAC
D L K R R L A D A V Q R S V G P A G Y W E S D D
1090 1100 1110 1120 1130 1140 1150
AACGACAACATGGGTACTCTGTCCCAGAACGCCAAGAAGTACCAGTCCTCCAACTCTGACCTGATCGCTGAC
N D N M G T L S Q N A K K Y Q S S N S D L I A D
1160 1170 1180 1190 1200 1210 1220
TTGGGTTTTGGTAAGGACGTCTACGGTGACGAGTGCTACCCTGGTGTCGTTGGTAAGTCTGCTATCTCTGAG
L G F G K D V Y G D E C Y P G V V G K S A I S E
1230 1240 1250 1260 1270 1280 1290
ACCTCCTACAGAGGTTTCTACAGAGCCTACCAGGCTCACATCTCCTCTTCCAACTGGGCTGAGTTCGAGAAC
T S Y R G F Y R A Y Q A H I S S S N W A E F E N

1300 1310 1320 1330 1340
ACCTCCAGAAACTGGCACACCGAACTCACCAAGACCACTGACAGATAA
T S R N W H T E L T K T T D R -

Done on DNA sequence DNTAdS
Total number of bases is: 585.
Analysis done on the complete sequence.
10 20 30 40 50 60 70
ATGATGATCAATACTCAGGAAGACAAGCTGGTTTCTGCTCATGATGCTGAAGAGTTCCACAGATTCTTCGTC
M M I N T Q E D K L V S A H D A E E F H R F F V
80 90 100 110 120 130 140
GGTCATGATTCTGACCTTCAGCAAGAAGTCACCACTCTTCTGACCAGAGAAGCTCACCTGCTGGACATCCAG
G H D S D L Q Q E V T T L L T R E A H L L D I Q
150 160 170 180 190 200 210
GCATACAAAGCCTGGCTGGAACACTGTGTCGCACCTGAGATCAAGTACCAAGTCATCTCCAGAGAACTGAGA
A Y K A W L E H C V A P E I K Y Q V I S R E L R
220 230 240 250 260 270 280
TCCACTTCCGAGAGAAGATACCAACTGAACGATGCTGTCAACATCTACAACGAGAACTACCAACAGCTGAAG
S T S E R R Y Q L N D A V N I Y N E N Y Q Q L K
290 300 310 320 330 340 350 360
GTCAGAGTCGAACACCAGATGGACCCACAGAACTGGTACAACTCTCCTAAGATCAGATTCACCAGATTCGTC
V R V E H Q M D P Q N W Y N S P K I R F T R F V
370 380 390 400 410 420 430
ACCAACGTCACTGCTGCTAAGGACAAGTCTGCTCCTGAGATGCTGCATGTCAGATCCAACCTGATCCTGCAC
T N V T A A K D K S A P E M L H V R S N L I L H
440 450 460 470 480 490 500
AGAGCCAGAAGAGGTAATCAAGTTGACGTCTTCTATGCCACCAGAGAAGACAAGTGGAAGAGAATCGAAGGT
R A R R G N Q V D V F Y A T R E D K W K R I E G
510 520 530 540 550 560 570
GGTGGTATCAAACTGGTCGAGAGATTCGTCGATTACCCTGAGAGATCCCCACAGACCCACAACCTGATCATC
G G I K L V E R F V D Y P E R S P Q T H N L I I
580
TTCCTGTAA
F L -

Done on DNA sequence DNTBS
Total number of bases is: 1683.
10 20 30 40 50 60 70
ATGCATCATGTTTCTACTAAGTCTCCATCTACCTTGTCTGCTGAATGTGAAGTTCTGATCGTCGGTGGTTCT
M H H V S T K S P S T L S A E C E V L I V G G S
80 90 100 110 120 130 140
CTGGTTGGTTTGTCTCTTGCTAACTTCCTTGGTCATCATGGTGTCTCTGCTGCTGTCGTCGAGAGACACAAG
L V G L S L A N F L G H H G V S A A V V E R H K

150 160 170 180 190 200 210

GGTACTGCCATTCATCCTAGAGCTGGTCACTTCCACCTGAGAACCATCGAAGCCTTCAGATACGCTGGTATC

G T A I H P R A G H F H L R T I E A F R Y A G I

220 230 240 250 260 270 280

GAGCCAGAAGTCATGCAGGAGTCCCTGAGACAGTTCGATCCTGATGGTGGTATCAACGTCGTCGAATCCCTG

E P E V M Q E S L R Q F D P D G G I N V V E S L

290 300 310 320 330 340 350 360

GCTGGTAAGGAGATTGCCTCTCTGATCGGTAATCTGAACGAAGGTGTCGAGAAGCTGTCTCCATCCAAGAGA

A G K E I A S L I G N L N E G V E K L S P S K R

370 380 390 400 410 420 430

CTGTTCATGACCCAACAGTCTCTGGAACCACTGCTGAGAAAGAACGCTGAGAAGCTGGGTGCTCAACTGAAC

L F M T Q Q S L E P L L R K N A E K L G A Q L N

440 450 460 470 480 490 500

TACCAGATGGAACTGGTCTCCTTCGAGCAGGATGCTACTGGTGTCACTGCTAGAGTCAGATACATCCCATCC

Y Q M E L V S F E Q D A T G V T A R V R Y I P S

510 520 530 540 550 560 570

GGTGCTGTCTCCCAAGTCAGAGCCAAGTACCTGATCGCTGCTGACGGTAATCGTTCTCCAGTCAGAGAGAAG

G A V S Q V R A K Y L I A A D G N R S P V R E K

580 590 600 610 620 630 640

CTGGGTATCGAGATGAGAGGTTACGGTTTGCTGTCCAACTCCATCACCATCTACTTCAAGGCTGACTGCACC

L G I E M R G Y G L L S N S I T I Y F K A D C T

650 660 670 680 690 700 710 720

AAGTGGATGGCTGGTAGAAACCTGGGTGTCGTCTACGTCAACAACCCTGACGTCAGAGGTTTCTTCAGACTG

K W M A G R N L G V V Y V N N P D V R G F F R L

730 740 750 760 770 780 790

ACCAGAGAGGCCAAGTCTGGTTTCCTGGGTGTCAACACCGTCGGTGATGTCTCCAGACCTGAGGCTAACAAC

T R E A K S G F L G V N T V G D V S R P E A N N

800 810 820 830 840 850 860

GTTGCTGAAGGTATCACTGCTGAGAGATGCGTCGAGATCGTCAGATCCGCTGTTGGTATCCCTGATCTGGAG

V A E G I T A E R C V E I V R S A V G I P D L E

870 880 890 900 910 920 930

GTTGAGATCGAGGGTATCGCTCCTTGGAGAGCTGTTGCTGACGTTGCTGACAGATACAGATCCGGAAACGTC

V E I E G I A P W R A V A D V A D R Y R S G N V

940 950 960 970 980 990 1000

TTCCTGATCGGTGATGCTGCACACGTCGTCCCACCAACTGGTGGTTTCGGTGGTAACACTGGTGTCCAGGAC

F L I G D A A H V V P P T G G F G G N T G V Q D

1010 1020 1030 1040 1050 1060 1070 1080

GCACACAACCTGGGTTGGAAGCTGGCTTCCGTCCTGAAGGGTCAAGCTGGTCCTGCTCTGCTGGACACCTAC

A H N L G W K L A S V L K G Q A G P A L L D T Y

1090 1100 1110 1120 1130 1140 1150

GAGGAGGAGAGAAGACCTGTCGGTCAACTGACCATCGAGCAGGCTTACTCCAGATACGTCCTGAGAATCGCA

E E E R R P V G Q L T I E Q A Y S R Y V L R I A

1160 1170 1180 1190 1200 1210 1220

CCTGAACTGGGTAGAGAGACCATGAAGCCTGTCGTCGATGACTTGTCTATGGAGATCGGTTACAGATACTTC

P E L G R E T M K P V V D D L S M E I G Y R Y F

1230 1240 1250 1260 1270 1280 1290

TCCTCTGCCATCTTGTCCAACGAGAAGAGAGGTGACAGAGTCTACGTTGACCCTAGAACCTCCTTCTCCTTG

S S A I L S N E K R G D R V Y V D P R T S F S L

1300 1310 1320 1330 1340 1350 1360

CCTGGTACTCGTGTTGGTCATCTGGTTTTCCAGAGAGATGGTAAGTCTATGGCAACCCTGGATGTCTGTGCT

P G T R V G H L V F Q R D G K S M A T L D V C A

1370 1380 1390 1400 1410 1420 1430 1440

GGTGGTATGACCCTGCTTGCTGGTGCTGGTGGTGTCGCTTGGTGCCAGTCCACCACTGAAGCTGCTGTCAAG

G G M T L L A G A G G V A W C Q S T T E A A V K

1450 1460 1470 1480 1490 1500 1510

CTGGGTATCGAGGTCGAGTCCAACGTCATCGGTAACGCTGGTGGTCTGACCGACGTCTCTGGTAGAGCACTG

L G I E V E S N V I G N A G G L T D V S G R A L

1520 1530 1540 1550 1560 1570 1580

GAGGTTCTGGGTATCGAGTCTGCTGGTGCCATTCTGGTCAGACCTGACGGTTTCGTCGCTTGGAGATCCGAG

E V L G I E S A G A I L V R P D G F V A W R S E

1590 1600 1610 1620 1630 1640 1650

CCTGGTGAGGCTGCTTCTGTCGCTAGAATGATCAACGTCCTGACCGCTGTCATGTGTCTGCAATCCCAGAGA

P G E A A S V A R M I N V L T A V M C L Q S Q R

1660 1670 1680

GTCGATGCCTCCGTCGTCGCTGCCTAA

V D A S V V A A -

Done on DNA sequence DNTDS

Total number of bases is: 945.

Analysis done on the complete sequence.

10 20 30 40 50 60 70

ATGGCTATCACTGACATTGCTTACTTGGTTAACGATCATACTGATCTTGAAGCTGCTGAGAGATTCTACACT

M A I T D I A Y L V N D H T D L E A A E R F Y T

80 90 100 110 120 130 140

GACTTCGGTTTGAAGGTTGCATACAGAACTGCTGACGAGATCGGTTTCAGACCTGCTCTTGCTAGAGGTTAC

D F G L K V A Y R T A D E I G F R P A L A R G Y

150 160 170 180 190 200 210

TGCTACGTCGCAAGAAAGTCCTCCAAGCCTGGTCTGAGAGCCATTGCCTTCACTGCCTCTACTCGTGCTGAC

C Y V A R K S S K P G L R A I A F T A S T R A D

220 230 240 250 260 270 280

CTGGATGTTGCTGCCAGATTCCCTGAGGCATCTCCAATCACTCCAATCGAGAGAGAAGGTGGTGGTGACAAG

L D V A A R F P E A S P I T P I E R E G G G D K

290 300 310 320 330 340 350 360

GTCATGCTTCAGTCTCCTGATGGTCTGCCATTCGAGATCGTTCATGGTATCCACTCCTACGAGGAACTGGCT

V M L Q S P D G L P F E I V H G I H S Y E E L A

370 380 390 400 410 420 430

GTCAGACCTGCTCTGACCTTCAACACTGGTAGAGTCAAGAGAAGACATGGTGAGTTCCAGAGACCACCACTG

```
 V  R  P  A  L  T  F  N  T  G  R  V  K  R  R  H  G  E  F  Q  R  P  P  L
     440       450       460       470       480       490       500
GAACCTGCACCAATCTTGAGACTTGGTCACGTTGCTCTGCTGACCAACGACTTCAAGAGAAACTTCGAGTGG
 E  P  A  P  I  L  R  L  G  H  V  A  L  L  T  N  D  F  K  R  N  F  E  W
   510       520       530       540       550       560       570
ATGCAGTCCAGACTGGGTCTGAGACCAACCGACACCATGTACGGTAAGGACAAGAACGACCTGGTTGGTTCC
 M  Q  S  R  L  G  L  R  P  T  D  T  M  Y  G  K  D  K  N  D  L  V  G  S
 580       590       600       610       620       630       640
TTCATGCACCTGTCTGGTGGTGGTGAGTGGACCGACCACCACTCCCTTGCCCTGTTCCCTGATCCATCTCCA
 F  M  H  L  S  G  G  G  E  W  T  D  H  H  S  L  A  L  F  P  D  P  S  P
650       660       670       680       690       700       710       720
AGAGTCCACCACTTCTCCTTCGAGGTCGAGGATCTCGATGCACAGGGTATGGGTAATCAGTGGCTGATCAAG
 R  V  H  H  F  S  F  E  V  E  D  L  D  A  Q  G  M  G  N  Q  W  L  I  K
        730       740       750       760       770       780       790
CATGGTTGGAAGCACACCTGGGGTATCGGTAGACACATCTACGGTTCTCAAATCTTCGACTACTGGTTCGAC
 H  G  W  K  H  T  W  G  I  G  R  H  I  Y  G  S  Q  I  F  D  Y  W  F  D
     800       810       820       830       840       850       860
CCTGATGGTAACATCGTCGAGCACTTCACTGATGGTGACTTGGTTCGTCCTGGTTGCAAGCCAGGTCTGGTC
 P  D  G  N  I  V  E  H  F  T  D  G  D  L  V  R  P  G  C  K  P  G  L  V
   870       880       890       900       910       920       930
GCTCTGGATGAGTCCCTGTTCGCTTGGGGTCCACCAATGCAGCCTGCCAACTTCGTCGAACTGGCTGCCCAC
 A  L  D  E  S  L  F  A  W  G  P  P  M  Q  P  A  N  F  V  E  L  A  A  H
 940
CACGAGTAA
 H  E  -
```

Done on DNA sequence DNTGS

Total number of bases is: 846.

Analysis done on the complete sequence.

```
         10        20        30        40        50        60        70
ATGAAATTGGCATCTTTCTTGTTGGATGGTAAGAGAAGATCCGGTGCATTGACTGATAAGGGTTTGGCTGTT
 M  K  L  A  S  F  L  L  D  G  K  R  R  S  G  A  L  T  D  K  G  L  A  V
       80        90       100       110       120       130       140
TTCGATGTTGATGGTGATATTGGTGATCTGCTGAGAAAGGGTACTGATCTTGCTGGTTTCGATGCACTGCTT
 F  D  V  D  G  D  I  G  D  L  L  R  K  G  T  D  L  A  G  F  D  A  L  L
   150       160       170       180       190       200       210
GCCAAGTCCTCCAGAATCGTTGATGCTGCCTCTGTCACCTTCGTCCAACCATCTGCTGAACCACCTAAAATC
 A  K  S  S  R  I  V  D  A  A  S  V  T  F  V  Q  P  S  A  E  P  P  K  I
 220       230       240       250       260       270       280
TTCTGTGTTGGTCTGAACTACGCTGACCACACTGCTGAGTCTCCATACGAACAGCCTGACTACCCAACCATC
 F  C  V  G  L  N  Y  A  D  H  T  A  E  S  P  Y  E  Q  P  D  Y  P  T  I
290       300       310       320       330       340       350       360
TTCTTGAGAGTCGCTACTGCTACCACTGGTCATGGTGCCTCTATCGCTCTGCCTGATCTGTCTGAGCAACTG
 F  L  R  V  A  T  A  T  T  G  H  G  A  S  I  A  L  P  D  L  S  E  Q  L
```

```
        370       380       390       400       410       420       430
GACTACGAAGGTGAGATGATCGCTGTCCTTGGTAAGGGTGGTAAGAGAATCCCTAAGGAGAGAGCCCATGAG
 D  Y  E  G  E  M  I  A  V  L  G  K  G  G  K  R  I  P  K  E  R  A  H  E
      440       450       460       470       480       490       500
CACGTCTTCGGTTACGCTGTCGGTAACGAGGTCTCTGTCAGAGACTACCAGTTCAAGTCTCCACAGTGGACC
 H  V  F  G  Y  A  V  G  N  E  V  S  V  R  D  Y  Q  F  K  S  P  Q  W  T
    510       520       530       540       550       560       570
ATTGGTAAGAACTTCGATGGTACTGGTGCTTGGGGTCCATACATCGTTACTGCTGACGAACTCCCACTTGGT
 I  G  K  N  F  D  G  T  G  A  W  G  P  Y  I  V  T  A  D  E  L  P  L  G
  580       590       600       610       620       630       640
GGACATGGTCTGAAGATCGAGGTCAGACTGAACGGTAAGACCGTCCAGTCCTCCAACACTGGTCACATGATC
 G  H  G  L  K  I  E  V  R  L  N  G  K  T  V  Q  S  S  N  T  G  H  M  I
650       660       670       680       690       700       710       720
TTCGATGTTGCCACCATCATCTCCACCATCTCCCAGGCCATCACCTTGCAGCCTGGTGACATCATCTTCACC
 F  D  V  A  T  I  I  S  T  I  S  Q  A  I  T  L  Q  P  G  D  I  I  F  T
        730       740       750       760       770       780       790
GGTACTCCTGCTGGTGTTGGTCTGGGTCACAAGCCACCACTGTGGATGAAGGATGGTGACAAGGTCGAGGTC
 G  T  P  A  G  V  G  L  G  H  K  P  P  L  W  M  K  D  G  D  K  V  E  V
      800       810       820       830       840
GAGATCGAGGGTATCGGTCTGCTGAAGAACTCCATCGCCAAGGAAGTCACCTAA
 E  I  E  G  I  G  L  L  K  N  S  I  A  K  E  V  T  -
```

Done on DNA sequence DNTES

Total number of bases is: 1527.

Analysis done on the complete sequence.

```
         10        20        30        40        50        60        70
ATGTCTGCTGTTAGAGAATCTCTTTCTGATCAAGTTCCAACTGTCAAACTTCTTATTGATGGTAAGTTTGTC
 M  S  A  V  R  E  S  L  S  D  Q  V  P  T  V  K  L  L  I  D  G  K  F  V
      80        90       100       110       120       130       140
GATTCCAACACCACTGAATGGCTGGATGACGTCAACCCTGCAACTCAGGCTGTTCTGTCTCGTGTCCCTATG
 D  S  N  T  T  E  W  L  D  D  V  N  P  A  T  Q  A  V  L  S  R  V  P  M
    150       160       170       180       190       200       210
GCAACTGCTGAAGAGGTCAATGCTGCTGTCGCTGCTGCTAAGCGTGCATTCGCATCTTGGCGTCATGCACCA
 A  T  A  E  E  V  N  A  A  V  A  A  A  K  R  A  F  A  S  W  R  H  A  P
  220       230       240       250       260       270       280
ATCGGTACTCGTGCTCGTATCTTCCTGAAGTACCAGCAGCTGATTCGTGAGAACATGGGTGAACTTGCTGCT
 I  G  T  R  A  R  I  F  L  K  Y  Q  Q  L  I  R  E  N  M  G  E  L  A  A
290       300       310       320       330       340       350       360
TTGCTGACTGCTGAGCAAGGTAAGACTCGTTTGGATGCTGAAGGTGACATCTTCCGTGGTCTGGAGGTCGTC
 L  L  T  A  E  Q  G  K  T  R  L  D  A  E  G  D  I  F  R  G  L  E  V  V
        370       380       390       400       410       420       430
GAACACGCTGCTGCTATCGGTAATCTGCAACTTGGTGAACTTGCCAACAACGTTGCCAAGATGGTTGATACC
```

E H A A A I G N L Q L G E L A N N V A K M V D T

440 450 460 470 480 490 500

TACACTCGTCTTCAGCCAATCGGTGTCTGTGCTGGTATCACTCCATTCAACTTCCCTGCAATGATTCCACTG

Y T R L Q P I G V C A G I T P F N F P A M I P L

510 520 530 540 550 560 570

TGGATGTTCCCAATGGCAATCGCATGTGGTAACACCTTCGTCCTGAAGCCATCTGAGCAAGATCCTATGGTC

W M F P M A I A C G N T F V L K P S E Q D P M V

580 590 600 610 620 630 640

ACTATGCGTCTTGTCGAACTTGCACTGGAGGCTGGTGTTCCATCTGGTGTTCTGAACGTCGTTCATGGTGGT

T M R L V E L A L E A G V P S G V L N V V H G G

650 660 670 680 690 700 710 720

GCACAGGTCGTTGACGCTATCTGCGATCATCCTGACATCAAGGCAATCTCCTTTGTTGGTTCCACTCGTGTC

A Q V V D A I C D H P D I K A I S F V G S T R V

730 740 750 760 770 780 790

GGTACTCATGTCTACAACCGTGCCACTCTGGCTGGTAAGCGTGTCCAGTGCATGATGGGTGCCAAGAACCAC

G T H V Y N R A T L A G K R V Q C M M G A K N H

800 810 820 830 840 850 860

GCTATCCTGATGCCTGACGCTAACCGTGAGCAGTCCCTGAACGCTATTGCTGGTGCTGCCTTCGGTGCTGCT

A I L M P D A N R E Q S L N A I A G A A F G A A

870 880 890 900 910 920 930

GGTCAGCGTTGCATGGCTCTGCCTGTCCTTGTCATGGTTGGTGAGGCACAACGTTGGCTGCCTGACTTGGTC

G Q R C M A L P V L V M V G E A Q R W L P D L V

940 950 960 970 980 990 1000

GCCAAGGCCAAGGCACTGACTGTCAACGCTGGTGACGCTGCTGGTGCTGACGTTGGTCCACTGATCTCTCCA

A K A K A L T V N A G D A A G A D V G P L I S P

1010 1020 1030 1040 1050 1060 1070 1080

TCTGCATGTGGTCGTGTTCGTGAACTGATTGGTGAGGGTGTTGAGGCTGGTGCTAAGCTCGAACTGGATGGT

S A C G R V R E L I G E G V E A G A K L E L D G

1090 1100 1110 1120 1130 1140 1150

CGTGCTGTCTCTGTCTCTGGTTACGAGAAGGGTAACTTCGTTGGTCCAACCATCTTCTCTGGTGTCAAGCCT

R A V S V S G Y E K G N F V G P T I F S G V K P

1160 1170 1180 1190 1200 1210 1220

GGAATGTCCATCTACGACATCGAAATCTTCGGTCCTGTCCTGTGTGTCATTGCTGCTGACAACCTGGATGAG

G M S I Y D I E I F G P V L C V I A A D N L D E

1230 1240 1250 1260 1270 1280 1290

GCAATCGAGATCATCAACGCTAACCCATCTGGTAACGGTACTGCCATCTTCACCCAGTCTGGTGCTGCTGCT

A I E I I N A N P S G N G T A I F T Q S G A A A

1300 1310 1320 1330 1340 1350 1360

CGTCGTTTCGAAGAGGACATCGACGTCGGTCAGGTCGGTATCAATGTTCCAATTCCTGTCCCTGTTCCAATG

R R F E E D I D V G Q V G I N V P I P V P V P M

1370 1380 1390 1400 1410 1420 1430 1440

TTCTCCTTCACTGGTTCTCGTGCTTCCAAACTGGGTGATCTGGGTCCATACGGTAAGCAGGTCATCATGTTC

F S F T G S R A S K L G D L G P Y G K Q V I M F

1450 1460 1470 1480 1490 1500 1510

TACACCCAGACCAAGACCATCACTGCACGTTGGTTCGATGATGCCTCCGTCCGTAAGAACGTCAACACCACC
Y T Q T K T I T A R W F D D A S V R K N V N T T
1520
ATCGCACTTGACTAA
I A L D -

Note S2. Primer sets used for qRT-PCR.

Locus	sense	Anti-sense
Rice actin	AAG,ATC,CTG,ACG,GAG,CGT,GGT,TAC	CTT,CCT,AAT,ATC,CAC,GTC,GCA,CTT,C
DntAa	GGT,CCT,GCT,GGT,CCT,GGT,CAC	GAC,ACC,GGA,AGG,GTA,GAA,TGC
DntAb	TAC,GCT,ACC,GAC,AAC,ACC,TGC	TCC,AGC,TTC,AGC,ATG,ACT,CTC
DntAc	GCT,GAC,TTG,GGT,TTT,GGT,AAG	TCT,GTC,AGT,GGT,CTT,GGT,GAG
DntAd	AAC,GTC,ACT,GCT,GCT,AAG,GAC	GTG,GGT,CTG,TGG,GGA,TCT,CTC
DntB	GGT,CTG,ACC,GAC,GTC,TCT,GGT	TTA,GGC,AGC,GAC,GAC,GGA,GGC
DntD	GGT,ATG,GGT,AAT,CAG,TGG,CTG	AGC,GAA,CAG,GGA,CTC,ATC,CAG
DntG	TTC,GAT,GTT,GCC,ACC,ATC,ATC	GGT,GAC,TTC,CTT,GGC,GAT,GGA
DntE	CGT,CGT,TTC,GAA,GAG,GAC,ATC	CTT,ACG,GAC,GGA,GGC,ATC,ATC

Table S1 Information on eight exogenous genes and their function

Gene	Length (bp)	Amino acid residues	function	Protein source (Gene bank accession no.)	organism
dntAa	987	328	2,4-DNT dioxygenase subunit A	AAL50024.1	*Burkholderia cepacia*
dntAb	315	104	2,4-DNT dioxygenase subunit B	AAL50022.1	*Burkholderia cepacia*
dntAc	1344	447	2,4-DNT dioxygenase subunit C	AAL50021.1	*Burkholderia cepacia*
dntAd	585	194	2,4-DNT dioxygenase subunit D	AAL50020.1	*Burkholderia cepacia*

dntB	1683	560	methylnitrocatechol (MNC) monooxygenase	AAL50019.1	*Burkholderia cepacia*
dntD	945	314	trihydroxytoluene (THT) oxygenase	AAF89669.1	*Burkholderia cepacia*
dntG	846	281	bifunctional isomerase/hydrolase	AAL50014.1	*Burkholderia cepacia*
dntE	1527	508	methylmalonate semialdehyde dehydrogenases	AAL50012.1	*Burkholderia cepacia*

Table S2 Enhanced ^{13}C abundance of citrate, succinate, and fumarate in the TCA cycle of DS_R1

	Succinate(atom% ^{13}C)	Fumarate(atom% ^{13}C)	Malate(atom% ^{13}C)	Citrate(atom% ^{13}C)
2h	0.6	0	0	0
6h	1.3	0.6	0.9	0
24h	3.2	2.3	3.3	2.7

Table S3. The bacterial community composition of enriched consortia at the genus level.

Table S4. The genera varied among samples cultivated WT and DS_R1 rice seedlings at the genus level.

Table S5. The genera varied among samples cultivated WT and DS_R2 rice seedlings at the genus level.

Table S6. The genera varied among uncontaminated soil (control) and samples cultivated WT rice seedlings at the genus level.

Table S7. The genera varied among uncontaminated soil (control) and samples cultivated DS_R1 rice seedlings at the genus level.

Table S8. The genera varied among uncontaminated soil (control) and samples cultivated DS_R2 rice seedlings at the genus level.
